# Silencing FAF2 mitigates alcohol-induced hepatic steatosis by modulating lipolysis and PCSK9 pathway

**DOI:** 10.1097/HC9.0000000000000641

**Published:** 2025-02-19

**Authors:** Nazmul Huda, Praveen Kusumanchi, Yanchao Jiang, Hui Gao, Themis Thoudam, Ge Zeng, Nicholas J. Skill, Zhaoli Sun, Suthat Liangpunsakul, Jing Ma, Zhihong Yang

**Affiliations:** 1 Department of Medicine, Division of Gastroenterology and Hepatology, Indiana University School of Medicine, Indianapolis, Indiana, USA; 2 Department of Infectious Diseases, Nanfang Hospital, Southern Medical University, Guangzhou, China; 3 Department of Surgery, Louisiana State University Health Science Center, New Orleans, Louisiana, USA; 4 Department of Surgery, John Hopkins University, Baltimore, Maryland, USA; 5 Department of Biochemistry and Molecular Biology, Indiana University School of Medicine, Indianapolis, Indiana, USA; 6 Department of Research, Roudebush Veterans Administration Medical Center, Indianapolis, Indiana, USA

**Keywords:** alcohol-associated liver disease, FAF2, lipolysis, liver steatosis

## Abstract

**Background:**

Chronic alcohol consumption leads to lipid accumulation, oxidative stress, cellular damage, and inflammation in the liver, collectively referred to as alcohol-associated liver disease (ALD). FAF2/UBXD8/ETEA (Fas-associated factor 2) is a ubiquitin ligase adaptor protein that plays a crucial role in the ubiquitin-mediated degradation of misfolded proteins in the endoplasmic reticulum. A recent genome-wide association study indicated an association between FAF2 and ALD; however, the exact contribution of FAF2 to ALD pathogenesis remains unclear.

**Methods:**

FAF2 was knocked down using AAV-delivered shRNA in C57/BL6 mice. Mice were subjected to a chronic-plus-single binge ethanol feeding (NIAAA) model. Nine hours after gavage, liver, blood, and other organs of interest were collected for gene expression and biochemical analyses.

**Results:**

We first observed a significant elevation in hepatic FAF2 protein expression in individuals with ALD and in mice subjected to an ethanol-binge model. Interestingly, knocking down FAF2 in the liver using adeno-associated virus serotype 8–delivered short hairpin RNA conferred a protective effect against alcohol-induced liver steatosis in ethanol-binged mice. Transcriptomic analysis revealed that differentially expressed genes were enriched in multiple lipid metabolism regulation pathways. Further analysis of transcription factors regulating these differentially expressed genes suggested potential regulation by SREBP1. Several SREBP1 target genes, including *Fasn, Scd1*, *Lpin1*, and *Pcsk9* (proprotein convertase subtilisin/kexin type 9), were dysregulated in the livers of ethanol-fed FAF2 knockdown mice. Additionally, Pcsk9 could be regulated through the FOXO3-SIRT6 pathway in the livers of ethanol-fed FAF2 knockdown mice, leading to increased liver low-density lipoprotein receptor expression and reduced plasma LDL cholesterol levels. Furthermore, FAF2 knockdown in mouse liver enhanced adipose triglyceride lipase lipolytic activity by upregulating the adipose triglyceride lipase activator, comparative gene identification-58, and downregulating the adipose triglyceridelipase transport inhibitor, Elmod2, contributing to the alleviation of liver steatosis.

**Conclusions:**

Our study uncovers a novel mechanism involving FAF2 in the pathogenesis of ALD.

## INTRODUCTION

Alcohol-associated liver disease (ALD) poses a significant global health burden, impacting millions of individuals worldwide due to chronic and excessive alcohol consumption.^1^,^2^ The condition encompasses a spectrum of liver abnormalities, beginning with hepatic steatosis, which can progress to more severe forms such as alcohol-associated hepatitis, characterized by inflammatory liver injury. If left unchecked, these conditions can lead to fibrosis or cirrhosis, ultimately resulting in liver failure.^3^,^4^ Currently, no universally accepted and effective therapeutic treatment exists for ALD, making an understanding of its underlying pathogenesis crucial for developing targeted interventions.^5^

Lipolysis is a fundamental biological process responsible for breaking down triglycerides (TG) into free fatty acids and glycerol, critical for energy production during fasting, physical activity, and metabolic stress.^6^ This process is particularly important in the context of liver steatosis, as it helps reduce fat accumulation in liver cells, thereby mitigating the risk of progressing to more severe liver conditions associated with ALD.^7^ The enzyme adipose triglyceride lipase (ATGL) plays a pivotal role in lipolysis, serving as the rate-limiting enzyme in TG breakdown. Its activity is essential for controlling lipid content within liver cells and preventing excessive fat buildup.^8^ However, ATGL requires comparative gene identification-58 (CGI-58) as a coactivator to enhance its lipolytic activity. CGI-58 facilitates efficient TG breakdown by ATGL, underscoring its critical role in the lipolytic process.^6^,^9^

A key regulator in this process is ELMO Domain Containing 2 (ELMOD2), which influences the transport and localization of ATGL to lipid droplets (LDs), where TG breakdown occurs.^10^ ELMOD2 orchestrates the activity of the adenosine diphosphate-ribosylation factor 1-coat protein complex 1, a system involved in vesicle trafficking and protein movement between cellular compartments. This transport system is crucial for ensuring that ATGL reaches LDs, facilitating effective lipolysis.^10^,^11^ The adenosine diphosphate-ribosylation factor 1-coat protein complex 1 system’s involvement in vesicle trafficking highlights its importance in cellular lipid metabolism and overall regulation of fat storage and breakdown within cells.^11^ Disruptions in these regulatory pathways can lead to abnormal fat accumulation in liver cells, contributing to the development and progression of liver steatosis and subsequent ALD.

A genome-wide association study and meta-analysis conducted by Schwantes-An et al^12^ identified a significant association between the Fas-Associated Factor Family Member 2 (*Faf2*) gene and the development of alcohol-associated cirrhosis. Fas Associated Factor 2, also known as UBXD8 or ETEA, encodes a protein that serves multiple cellular functions, particularly in the endoplasmic reticulum (ER) and LDs.^13^ This protein is implicated in various processes, including protein degradation, apoptosis, and functioning as a fatty acid sensor.^14^,^15^ Research by Olzmann et al^13^ revealed that FAF2 directly interacts with ATGL, a crucial enzyme in the lipolysis pathway responsible for breaking down TGs into free fatty acids and glycerol within LDs. This interaction suggests that FAF2 may play a regulatory role in lipolysis by influencing ATGL activity.

FAF2 also plays a crucial role in the ER-associated degradation pathway, particularly in the degradation of Insulin-induced gene 1 (INSIG-1).^16^ INSIG-1 is a protein that retains the SREBP cleavage-activating protein/Sterol regulatory element-binding protein complex in the ER under normal conditions.^17^,^18^ When INSIG-1 is degraded, it releases the SREBP cleavage-activating protein/Sterol regulatory element-binding protein complex, allowing it to translocate to the Golgi apparatus.^19^ There, the complex undergoes cleavage, activating SREBPs, especially SREBP1, a key transcriptional regulator of genes involved in fatty acid synthesis.^20^,^21^ The activation of SREBP1 through this pathway enhances the expression of genes involved in fatty acid synthesis, including those encoding enzymes necessary for lipid biosynthesis and storage.^21^,^22^ By promoting the release and activation of SREBP1, FAF2 indirectly supports the upregulation of lipogenic genes, contributing to increased fatty acid synthesis.

The objective of this study was to determine the role of FAF2 in the pathogenesis of ALD. Understanding FAF2’s role in this context will provide insights into the molecular mechanisms driving hepatic steatosis, potentially informing the development of targeted therapies aimed at modulating FAF2 activity. This knowledge could offer new strategies for preventing and treating ALD.

## METHODS

### Animal experiments

The Institutional Animal Care and Use Committee at Indiana University School of Medicine approved all animal experiments. Male C57BL/6J mice (wild type), aged 6–8 weeks and weighing approximately 20–25 g, were obtained from Jackson Laboratory (Bar Harbor, ME). On arrival, the mice were housed under a 12-hour light/12-hour dark cycle and allowed to acclimatize for 1 week before the experiments began. Mice were randomly assigned to either a control diet (F1259SP, Bio-Serv, Flemington, NJ) or an ethanol-containing diet (F1258SP, Bio-Serv, Flemington, NJ). The ethanol concentration in the diet was gradually increased by 1% (vol/vol) daily until it reached 5% on day 5, which was then maintained for the subsequent 10 days. Daily consumption was monitored, and any remaining diet was replenished with freshly prepared food accordingly. On the 16th day, mice on the ethanol-containing diet received a single oral dose of ethanol via gavage at a dose of 5 g/kg body weight. Mice in the control group received an equivalent volume of maltose solution via gavage. After a 9-hour postgavage period to allow for ethanol metabolism, the mice were euthanized, and blood, liver, and other relevant tissues were promptly collected and snap-frozen in liquid nitrogen for subsequent analysis.

### Construction of targeting vector for FAF2 knockdown

In this study, *Faf2* short hairpin RNA (shRNAs) and a control shRNA (Supplemental Table S1, http://links.lww.com/HC9/B884) were cloned into the pAAV-ZsGreen-shRNA vector obtained from Creative Biogene. The *ZsGreen* gene was driven by a cytomegalovirus promoter, while the shRNA was driven by a U6 promoter. Cloning was performed at the BamHI-HindIII site using standard molecular biology techniques, and plasmid preparations followed standard protocols. adeno-associated virus serotype (AAV) subtype 8 viral particles carrying the pAAV-*ZsGreen-Faf2*-shRNA or control shRNA plasmids were generated as described.^23^,^24^ The purified AAV viral particles, at a dose of 5×10^11^ viral particles/mouse, were administered to mice via tail vein injection 2 weeks prior to the initiation of alcohol feeding. In vitro knockdown of FAF2 was performed using the pAAV-*ZsGreen-Faf2*-shRNA in the presence or absence of ethanol at a defined concentration in VL-17A or AML-12 cell lines.

The *Flag-Faf2* recombinant plasmid was constructed by inserting mouse *Faf2* into NotI-NcoI site of pcDNA6-3xFLAG (Addgene, MA) vector by Synbio-Tech (Monmouth Junction, NJ). The recombinant plasmid was used to transfect AML12 in order to study exogenous FAF2 expression.

### RNA sequencing

RNA sequencing was performed on liver RNA samples obtained from ethanol-fed *Faf2* knockdown mice (n=4) and control shRNA mice (n=4), ensuring that the RNA integrity number was >7, in accordance with standard protocols.^25^ The sequencing was outsourced to LC Sciences (Houston, TX). Poly(A) RNA sequencing libraries were prepared following Illumina’s TruSeq stranded-mRNA sample preparation protocol and paired-end sequencing was conducted on Illumina’s NovaSeq. 6000 sequencing system. Data preprocessing included adapter removal, trimming low-quality bases, and filtering out undetermined bases using Cutadapt and custom Perl scripts. The reads were then mapped to the mouse genome using HISAT2. Transcript assembly and quantification were performed with StringTie and Ballgown to estimate transcript expression levels in fragments per kilobase of transcript per million mapped reads. Differential expression analysis was conducted using DESeq. 2, with significance thresholds set at a false discovery rate of <0.05 and absolute fold change (|FC|) of 2 or greater (−1>log2(FC)>1). Visualization of differential expression results was accomplished using R packages to generate volcano plots, heatmaps, and dot plots. Pathway enrichment analysis was performed using Gene Set Enrichment Analysis (GSEA) software.^26^ The prediction of transcription factors regulating the differentially expressed genes (DEGs) was conducted using Transcriptional Regulatory Relationships Unraveled by Sentence-based Text mining in the Enrichr platform (https://maayanlab.cloud/Enrichr/).^27^ The raw sequencing data were deposited in the NCBI Gene Expression Omnibus under accession number GSE270659. The primer sequences used for quantitative polymerase chain reaction (qPCR) are listed in Supplemental Table S2, http://links.lww.com/HC9/B884.

### Immunohistochemistry and immunofluorescence staining

Liver tissues were fixed in 10% formalin, embedded in paraffin, and sectioned into 5 µm thick slices. Hematoxylin and eosin staining was performed according to standard protocols for histological examination. Ag retrieval was achieved by heating sections in citrate buffer (pH 6.0) at 95 °C for 20 minutes after deparaffinization and rehydration. Diluted primary antibodies specific to FAF2, MPO, and F4/80 (as detailed in Supplemental Table S3, http://links.lww.com/HC9/B884) were applied to the sections and incubated overnight at 4 °C. Images were scanned with an Aperio CS2 scanner (Leica, IL). For immunofluorescence studies using paraffin-embedded sections, the protocol was similar until the secondary antibody step, where horseradish peroxidase-labeled secondary antibodies were replaced with fluorochrome-conjugated secondary antibodies. Nuclei were counterstained with DAPI before mounting (Prolong Diamond Antifade Mountant with DAPI, Invitrogen, Cat#P36966). For immunofluorescence staining on cultured cells, the cells were fixed with 4% paraformaldehyde for 20 minutes and blocked with 1xPBS containing 1% BSA, 0.01% Triton X-100 for 30 minutes before incubating overnight at 4 °C with primary antibodies of interest (Supplemental Table S3, http://links.lww.com/HC9/B884). After washing, cells in the slide were incubated with secondary antibodies of interest labeled with fluorescent dyes (Supplemental Table S3, http://links.lww.com/HC9/B884). The LDs were stained with either BODIPY (Sigma-Aldrich Inc.) or LipidSpot (Biotium, Fremont, CA). Imaging was obtained by a confocal microscope (Leica TCS-SP8, Leica Microsystems Inc.).

### Hepatic LD isolation

LDs were isolated from liver tissues following the manufacturer’s instructions (Cat# MET-5011, Cell Biolabs Inc., San Diego, CA). Approximately 50 mg of liver tissue was placed in a prechilled glass Dounce tissue grinder (Wheaton, IL). First, 0.2 mL of solution A from the kit was added, and the mixture was incubated on ice for 10 minutes. Following this, 0.8 mL of solution B was added, and the mixture was further incubated on ice for another 10 minutes. The liver tissues were then lysed using a loose pestle followed by a tight pestle, with 5 strokes each. After lysing, the homogenates were transferred to a 2 mL tube, and 0.6 mL of solution B was carefully layered on top of the lysates. The sample was then centrifuged at 20,000*g* for 3 hours at 4 °C. After centrifugation, 0.27 mL of the floating layer containing LD was collected into a fresh tube. The isolated LD fraction was normalized based on protein content, determined using the pierce bicinchoninic acid assay protein assay reagent (Thermo-Fisher Scientific, Waltham, MA). This normalized LD fraction was subsequently used for TG analysis, lipase activity assays, and western blot analysis. Any remaining LDs were stored at −80 °C until further use.

### Supplementary methods

The methods for the cell culture and in vitro ethanol treatment, qPCR, immunoblotting, mouse primary hepatocyte isolation, and biochemical analysis were provided in Supplemental Materials, http://links.lww.com/HC9/B885.

### Statistical analysis

The data are presented as mean±SEM and were analyzed using GraphPad Prism 9.5 (GraphPad Software, San Diego, CA). To assess statistical significance, either a Student *t* test or a one-way ANOVA with Tukey post hoc test for multiple comparisons was conducted, depending on the specific circumstances.

## RESULTS

### Ethanol induces expression of FAF2 in both mouse and human livers

**FIGURE 1:**
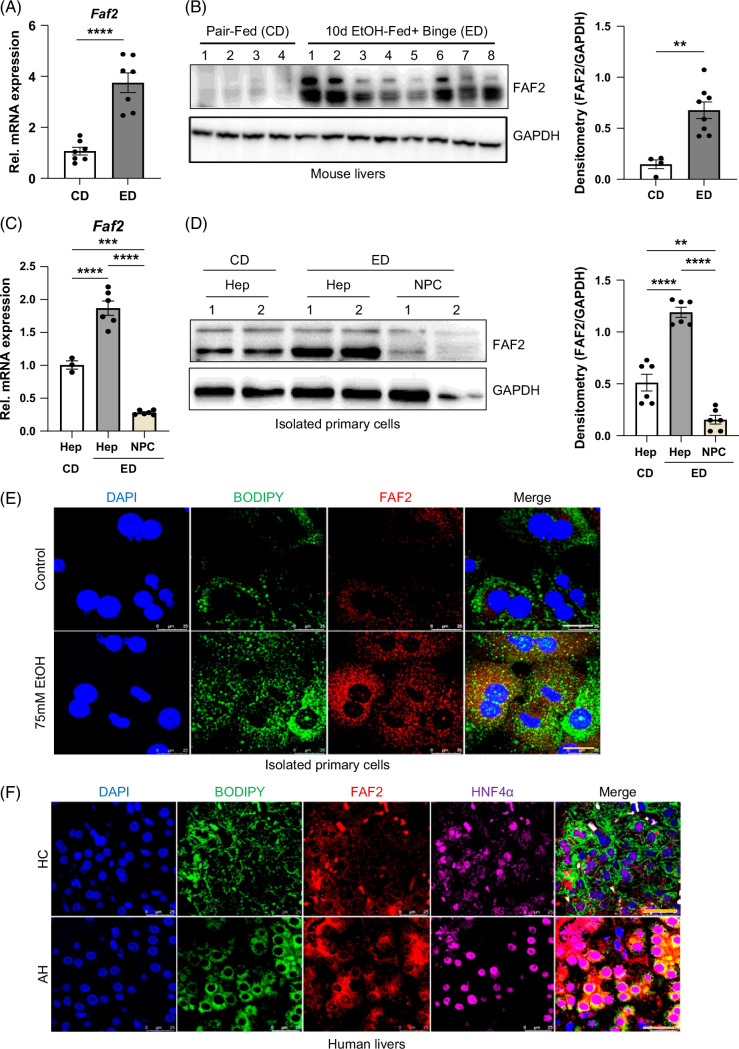
Ethanol induces FAF2 expression in the liver. (A) Quantitative PCR analysis of relative *Faf2* mRNA expression in the liver of pair-fed and ethanol-fed mice. (B) Western blot analysis (left) and densitometric quantification (right) of FAF2 protein levels in the liver of pair-fed and ethanol-fed mice. (C) Relative *Faf2* mRNA expression in isolated primary hepatocytes (Hep) and NPC from pair-fed (CD) and ethanol-fed (ED) mice. (D) Representative western blot images (left) and densitometric quantification (right) of FAF2 protein in isolated Hep and NPC from pair-fed (CD) and ethanol-fed (ED) mice. (E) Representative immunofluorescence images of isolated primary hepatocytes from control diet-fed mice treated with ethanol in vitro with FAF2 antibody (red) and BODIPY (lipid droplets, green). Scale bar: 25 µm. (F) Representative immunofluorescence images of liver sections from healthy controls (HC) and patients with alcoholic hepatitis (AH), stained with FAF2 antibody (red), HNF4α antibody (purple), BODIPY for lipid droplets (green), and DAPI for nuclei (blue). Scale bar: 25 µm. Data are presented as mean±SEM, with *p-*values indicated on bar graphs for comparisons with respective controls. **p<0.01; ***p<0.001; ****p<0.0001 versus the indicated group. Abbreviations: AH, alcoholic hepatitis; CD, control diet; ED, ethanol-containing diet; EtOH, ethanol; FAF2, Fas-associated factor 2; NPC, nonparenchymal cell.

Mice subjected to an ethanol diet for 10 days, followed by a single binge, exhibited significant upregulation of *Faf2* mRNA and protein expression in the liver compared to pair-fed control mice (Figure 1A, B, and Supplemental Figure S1A, http://links.lww.com/HC9/B886). FAF2 expression was primarily localized to hepatocytes rather than nonparenchymal cells (Figure 1C, D and Supplemental Figures S1B, C, http://links.lww.com/HC9/B886). Mouse primary hepatocytes and the AML12 cell line treated with ethanol also demonstrated increased FAF2 expression, corroborating the in vivo findings (Figure 1E and Supplemental Figures S1B–E, http://links.lww.com/HC9/B886). Additionally, the human hepatic cell line VL-17A, a recombinant HepG2 cell line that expresses both alcohol dehydrogenase (ADH) and cytochrome P450 2E1(CYP2E1), displayed elevated FAF2 expression upon ethanol treatment (Supplemental Figure S1F, G, http://links.lww.com/HC9/B886). Notably, ethanol treatment did not affect the subcellular localization of FAF2 (Supplemental Figures S2A–C, http://links.lww.com/HC9/B886). Liver tissue sections from patients with alcohol-associated hepatitis revealed higher FAF2 expression in HNF4α-positive cells compared to healthy controls, as shown in Figure 1F. Collectively, these data indicate that ethanol consumption induces a significant upregulation of FAF2 expression in both mouse and human livers, particularly in hepatocytes.

### Liver-specific knocking down of *Faf2* has a protective effect against ethanol-induced hepatic steatosis in mice

**FIGURE 2:**
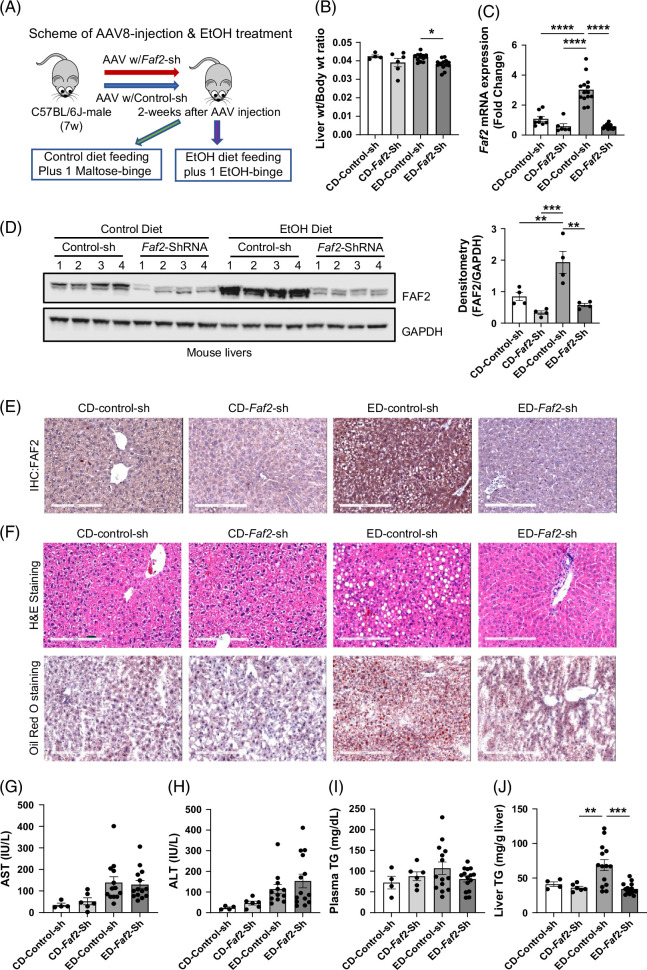
Liver-specific knockdown of FAF2 ameliorates alcohol-induced steatosis in mice liver. (A) Schematic diagram illustrating the tail vein injection strategy used to deliver AAV8-control shRNA or AAV8-*Faf2*-shRNA into mouse livers, followed by ethanol feeding. (B, C) Liver-to-body weight ratio (B) and relative mRNA expression of *Faf2* (C) in CD and ED groups, with mice harboring either control shRNA or *Faf2*-shRNA. (D, E) Representative western blot analysis (D) and IHC images (E) demonstrating the efficiency of AAV-mediated knockdown of FAF2 protein in the livers of mice fed with either control or ethanol diet. (F) Representative images of H&E staining (top) and Oil Red O staining (bottom) in the livers of mice on control or ethanol diets. (G–K) Plasma levels of AST (G), ALT (H), triglycerides (I), liver triglycerides (J), and total liver cholesterol (K) in mice expressing either control shRNA or *Faf2*-shRNA and fed with either control or ethanol diet. Scale bar: 200 µm. Data are presented as mean±SEM, with *p-*values indicated on bar graphs for comparisons with respective controls. **p<0.01; ***p<0.001; ****p<0.0001 versus the indicated group. Abbreviations: AAV8, adeno-associated virus serotype 8; CD, control diet; ED, ethanol-containing diet; EtOH, ethanol; FAF2, Fas-associated factor 2; H&E, hematoxylin and eosin; IHC, immunohistochemistry; shRNA, short hairpin RNA; TG, triglyceride.

To investigate the role of FAF2 in ALD, we employed shRNA to suppress its expression. Among the three shRNAs tested (Supplemental Table S1, http://links.lww.com/HC9/B884), shRNA #3 proved to be the most effective in vitro for knocking down FAF2 expression (Supplemental Figure S3A–C, http://links.lww.com/HC9/B886). Adeno-associated virus subtype 8 (AAV8), carrying either a scrambled shRNA (control) or *Faf2*-specific shRNA #3, was administered via tail vein injection into the livers of C57BL/6J mice (Figure 2A). After 2 weeks, the mice were fed either a control or an ethanol-containing diet for 10 days, followed by a binge of maltose (control) or ethanol (Figure 2A). Ethanol-fed mice with *Faf2* knockdown exhibited a significant reduction in the liver-to-body weight ratio compared to ethanol-fed control mice (Figure 2B). This knockdown led to a significant decrease in *Faf2* mRNA and protein levels in the livers of ethanol-fed mice (Figure 2C, D), while no changes were observed in adipose tissue (Supplemental Figures S3D, http://links.lww.com/HC9/B886). Notably, *Faf2* knockdown in ethanol-fed mice resulted in a marked reduction in hepatic steatosis, as evidenced by decreased intracellular LD accumulation in hematoxylin and eosin and Oil Red O staining (Figure 2E, F).

However, ethanol-induced elevations in serum ALT and AST were not significantly affected by *Faf2* knockdown (Figure 2G, H), nor were there any significant changes in liver inflammation markers (eg, TNFα, IL1b, IL6) or in the number of infiltrating neutrophils and macrophages (Supplemental Figure S4A–D, http://links.lww.com/HC9/B886). Interestingly, liver TG levels were significantly reduced in ethanol-fed *Faf2* knockdown mice, while plasma TG levels showed no significant change (Figure 2I-J). Additionally, total cholesterol levels in mice livers with *Faf2* knockdown also did not exhibit significant alterations (Supplemental Figures S4E, http://links.lww.com/HC9/B886).

### Identification of DEGs in the livers of ethanol-fed mice with *Faf2* knockdown

**FIGURE 3:**
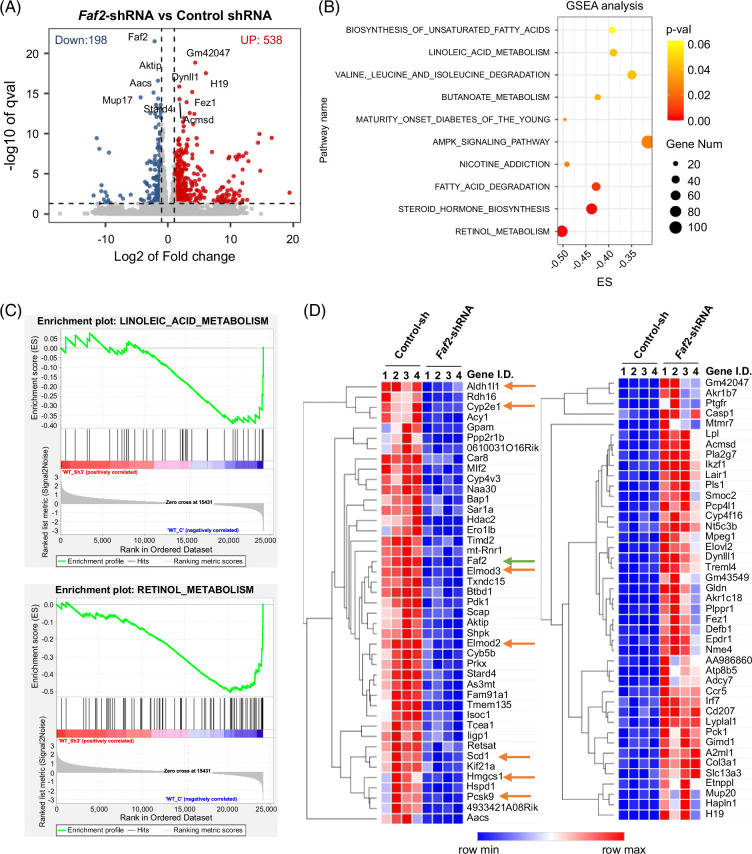
RNA-sequencing data suggest that FAF2 regulates lipid metabolism in the liver. (A) Volcano plot illustrating the differentially expressed genes identified through RNA sequencing. Blue and red dots indicate downregulated and upregulated genes, respectively, based on an adjusted *p*-value (qval)<0.05 and −1>log2 (FC)>1. The top 10 genes ranked by qval are labeled. (B) GSEA of the differentially expressed genes, presented in a dot plot format for the top 10 enriched pathways. The size of each dot represents the number of genes involved, while the color indicates the *p-*values. (C) Enrichment plots for the Linoleic acid metabolism and retinol metabolism pathways, illustrating the significance of these pathways in the analysis. (D) Heatmap analysis showing the top 100 genes that significantly changed between the control and *Faf2*-specific shRNA knockdown groups. Downregulated and upregulated genes are displayed separately, with red arrows indicating genes involved in lipid metabolism and a green arrow highlighting the *Faf2* gene. Color coding is based on log-transformed read count values. Abbreviations: ES, enrichment score; FAF2, Fas-associated factor 2; GSEA, Gene Set Enrichment Analysis; shRNA, short hairpin RNA.

To understand the mechanism underlying the protection against alcohol-induced hepatic lipid accumulation conferred by FAF2 deficiency, we performed RNA sequencing analysis on liver samples from mice fed an ethanol diet, comparing control and *Faf2* knockdown groups. Subsequent bioinformatics approaches aimed to uncover the molecular mechanisms associated with the impact of *Faf2* knockdown on the pathogenesis of ALD. DEGs were visualized using volcano plots, revealing 538 upregulated and 198 downregulated genes in the *Faf2* knockdown group compared to controls (Figure 3A). Gene Set Enrichment Analysis (GSEA) was conducted to identify enriched pathways associated with *Faf2* knockdown. The top 10 enriched pathways predominantly involved fatty acid metabolism, including linoleic acid metabolism, retinol metabolism, fatty acid degradation, biosynthesis of unsaturated fatty acids, and the AMPK signaling pathway (Figure 3B and Supplemental Figures S5A–C, http://links.lww.com/HC9/B886). Enrichment plots demonstrated negatively enriched gene sets in the linoleic acid metabolism and retinol metabolism pathways, indicating that *Faf2* knockdown downregulated these pathways (Figure 3C). Notably, key genes such as *Cyp4a10 and Cyp4a14*, previously proved to be associated with ALD, were among those downregulated and enriched in the retinal metabolism pathway (Supplemental Figure S5A, http://links.lww.com/HC9/B886).^28^ Heatmap analysis of the AMPK signaling pathway revealed significant changes in genes regulating lipid metabolism, including *Acaca, Acacb, Srebf1, Lepr, Fasn, and Scd1* (Supplemental Figure S5B, http://links.lww.com/HC9/B886). Heatmaps illustrating the most significantly downregulated (Figure 3D, left) and upregulated (Figure 3D, right) genes highlighted *Faf2* as one of the significantly downregulated genes (Figure 3D, green arrow). Genes associated with lipid and ethanol metabolism, such as *Scd1, Hmgcs1, Aldh1l1, Cyp2e1, and Pcsk9*, were also observed to be downregulated in the *Faf2* knockdown livers (Figure 3D). Both Gene Ontology term analysis using the R program and GSEA analysis with Gene Ontology pathways indicated that DEGs were significantly enriched in lipid metabolic processes, suggesting a dysregulation of lipid metabolism pathways (Supplemental Figures S5C, http://links.lww.com/HC9/B886 and S6A–C, http://links.lww.com/HC9/B886). Collectively, these findings suggest that *Faf2* knockdown exerts a deregulatory effect on lipid metabolism pathways and gene expression, contributing to reduced alcohol-induced hepatic lipid accumulation.

### *Faf2* knockdown reduced SREBP1 expression and downstream target genes

**FIGURE 4:**
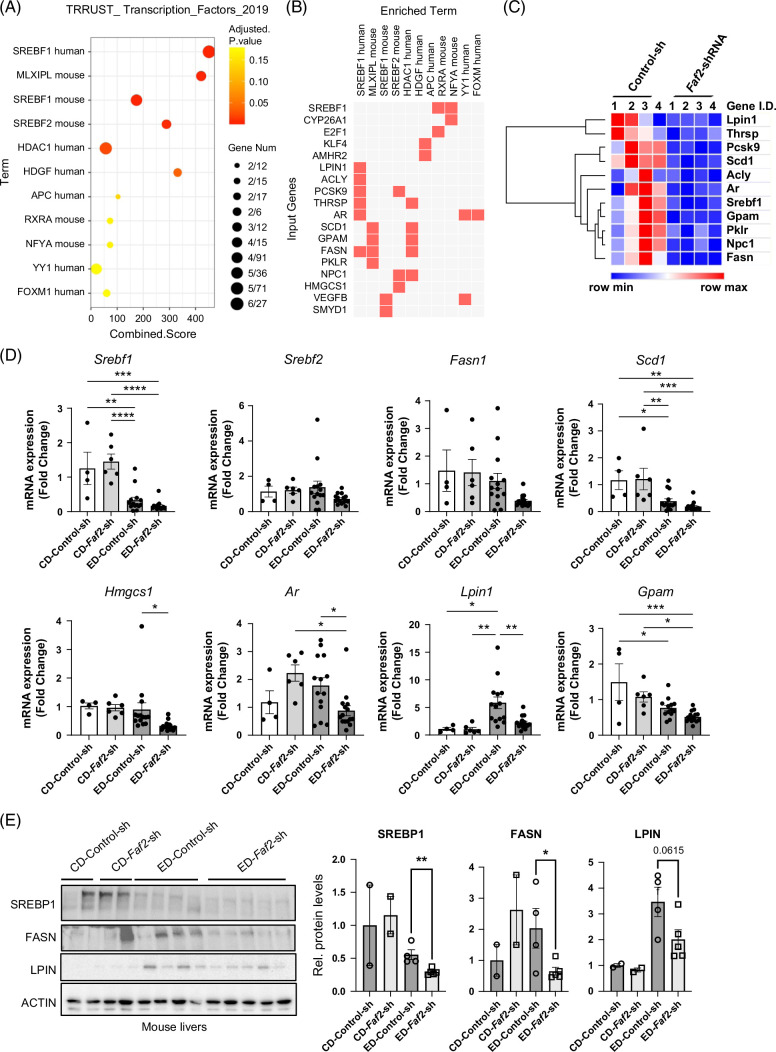
FAF2 regulates lipid metabolism in the liver through SREBP1. (A) Dot plot illustrating predictions of transcription factors that regulate the differentially expressed genes, utilizing Transcriptional Regulatory Relationships Unraveled by Sentence-based Text mining database. (B) Clustergram displaying individual genes enriched in the predicted transcription factor terms, highlighting specific regulatory relationships. (C) Heatmap of the genes identified in (B) based on RNA sequencing analysis, showing expression patterns across different samples. (D) Quantitative PCR analysis of selected genes from (C), with each dot representing an individual mouse liver. (E) Western blot analysis of SREBP1, FASN, and LPIN protein levels in mouse livers from the indicated groups. Each line corresponds to an individual mouse liver, with densitometric analysis provided. ACTIN (beta-ACTIN) was used as the loading control. Statistical significance is indicated as *p<0.05; **p<0.01; ***p<0.001 compared to the indicated group. Abbreviations: CD, control diet; CYP2E1, cytochrome P450 2E1; ED, ethanol-containing diet; FAF2, Fas-associated factor 2; shRNA, short hairpin RNA; SREBP1, Sterol Regulatory Element Binding Protein 1.

To elucidate the regulatory mechanisms affected by *Faf2* knockdown, we examined potential transcription factors governing the DEGs using the Transcriptional Regulatory Relationships Unraveled by Sentence-based Text mining database. Remarkably, SREBF1 emerged as 1 of the top 10 transcription factors, identified by its highest score and lowest adjusted *p-*values (Figure 4A). Clustergram demonstrated that the genes enriched as targets of SREBF1 transcriptional regulation included *Lpin1, Fasn, Acly, Ar, and Pcsk9* (Figure 4B). Specifically, the expression heatmap (Figure 4C) indicated a clear reduction of those gene expressions in the livers of ethanol-fed *Faf2* knockdown mice compared to those with control shRNA. To validate these findings, qPCR confirmed the downregulation of these SREBF1 target genes (Figure 4D). Consistent with the reduced mRNA levels of *Srebf1*, western blot demonstrated a significant decrease in the protein levels of SREBP1 in the livers of ethanol-fed *Faf2* knockdown mice compared to ethanol-fed control groups (Figure 4E and Supplemental Figure 7A, B, http://links.lww.com/HC9/B886). Correspondingly, protein levels of FASN and LPIN, downstream targets of SREBP1, were also reduced in parallel with the decrease in SREBP1 in the livers of ethanol-fed *Faf2* knockdown mice (Figure 4E and Supplemental Figure S7A, B, http://links.lww.com/HC9/B886). These findings indicate that *Faf2* knockdown regulates SREBP1 and its targets, thereby reducing fatty acid synthesis and lipid accumulation under conditions of alcohol feeding.

### The upregulation of LDLR in mouse liver and the reduction of PCSK9 levels in both liver and plasma as a result of FAF2 knockdown

**FIGURE 5:**
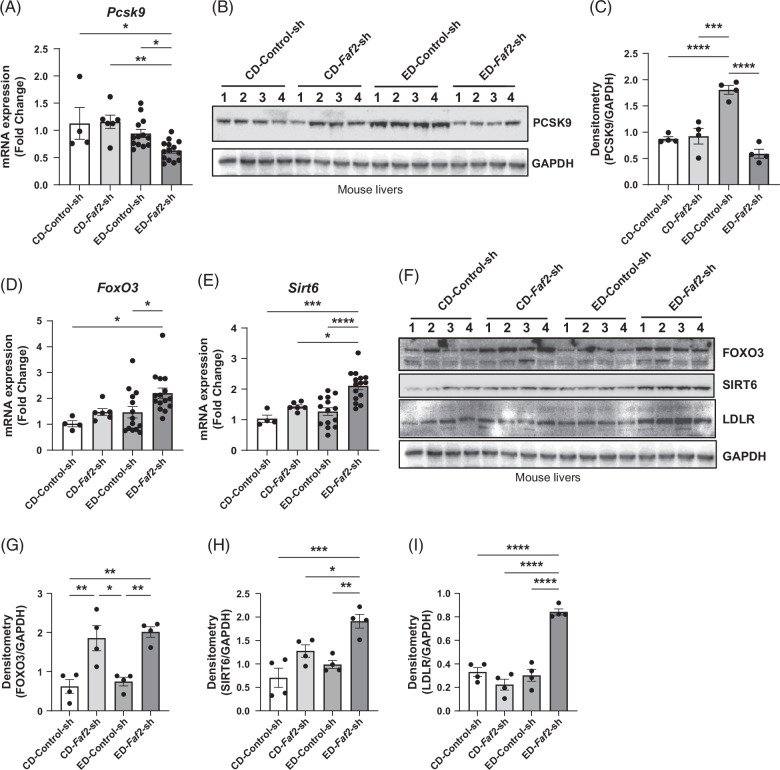
Knocking down of *Faf2* caused a significant reduction of PCSK9 expression in mouse liver. (A–C) Relative mRNA expression (A) and protein levels (B, C) of PCSK9 in mouse livers expressing either control shRNA or *Faf2*-shRNA, under both control and ethanol diet conditions. (D) Relative mRNA expression of FOXO3 and SIRT6 in the indicated groups. (E, F) Representative western blot images displaying protein expression levels of FOXO3, SIRT6, PCSK9, LDLR, and GAPDH (loading control) in the livers of mice expressing either control shRNA or *Faf2*-shRNA, fed with either control or ethanol diet. (G–I) Densitometric analysis of FOXO3, SIRT6, and LDLR protein levels, with GAPDH serving as the loading control. Each dot represents an individual mouse liver sample. Data are presented as mean±SEM, with *p-*values compared to respective controls shown in the bar graphs. Statistical significance is indicated as *p<0.05; **p<0.01; ***p<0.001; ****p<0.0001 compared to the indicated group. Abbreviations: CD, control diet; ED, ethanol-containing diet; FAF2, Fas-associated factor 2; LDLR, low-density lipoprotein receptor; PCSK9, proprotein convertase subtilisin/kexin type 9.

**FIGURE 6:**
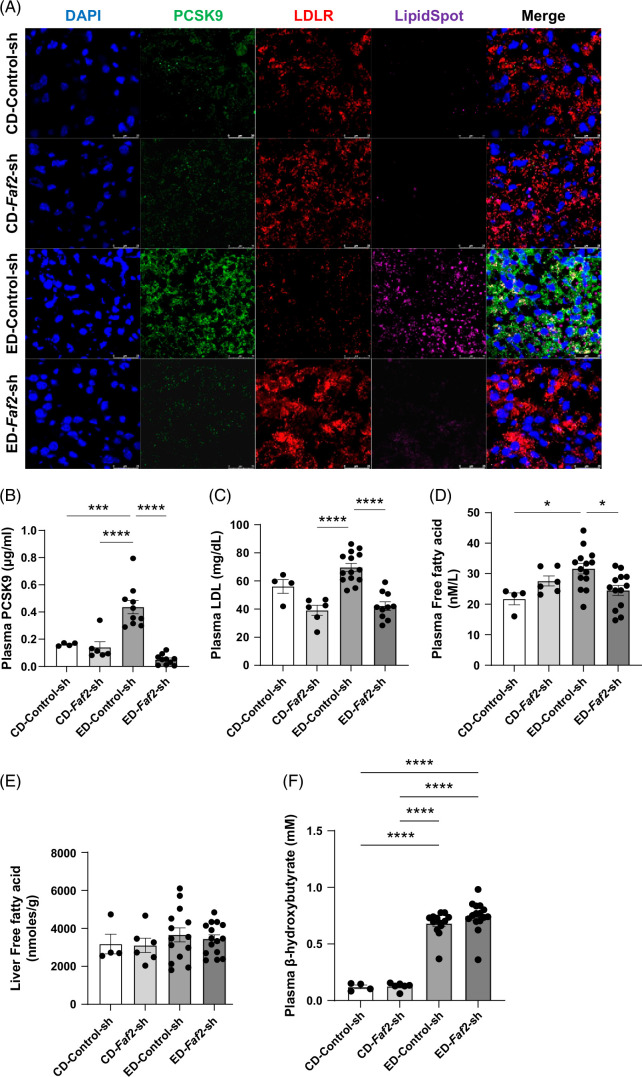
Knocking down of *Faf2* caused a significant reduction of plasma PCSK9, LDL, and free fatty acid levels. (A) Representative immunofluorescence images showing relative expression of PCSK9 (green) and LDLR (red) in liver sections from *Faf2* knockdown and control groups. Nuclei and lipid droplets are stained with DAPI (blue) and LipidSpot 610 (purple). Scale bar: 25 µm. (B, C) Plasma levels of PCSK9 (B) and LDL-C (C) were measured by ELISA. (D–F) Plasma and liver free fatty acid levels were assessed colorimetrically, along with plasma β-hydroxybutyrate levels (F). Scale bar: 25 µm. Data are presented as mean±SEM, with *p-*values compared to respective controls shown in the bar graphs. Statistical significance is indicated as *p<0.05; **p<0.01; ***p<0.001; ****p<0.0001 compared to the indicated group. Abbreviations: CD, control diet; ED, ethanol-containing diet; FAF2, Fas-associated factor 2; LDLR, low-density lipoprotein receptor; PCSK9, proprotein convertase subtilisin/kexin type 9.

Our RNA sequencing analysis revealed a significant downregulation of PCSK9 in the livers of ethanol-fed mice with *Faf2* knockdown compared to the control group (Figures 3D and 4B, C). Previous studies have suggested that PCSK9 deficiency confers resistance to hepatic steatosis.^29^,^30^ To validate these findings, we conducted qPCR and western blot analyses, confirming a substantial reduction in PCSK9 expression in the *Faf2*-knockdown group (Figure 5A–C). It has been reported that FOXO3 and SIRT6 can inhibit the PCSK9 expression.^31^ In our study, we observed a significant upregulation of FOXO3 and SIRT6 at both the mRNA and protein levels, which could contribute to the reduction of PCSK9 in ethanol-fed *Faf2*-knockdown livers (Figure 5D–I). PCSK9 plays a critical role in regulating LDLR turnover, thereby influencing the balance of low-density lipoprotein cholesterol (LDL-C) in the body. Consistent with the reduced PCSK9 levels, we observed an increase in LDLR protein levels in *Faf2* knockdown livers, confirmed by both western blotting and immunofluorescence (Figures 5F and 6A). Furthermore, we investigated the impact of *Faf2* knockdown on circulating PCSK9 and LDL-C in plasma and found significant reductions in both parameters in ethanol-fed *Faf2* knockdown mice compared to controls (Figure 6B, C). Additionally, plasma free fatty acids were reduced in ethanol-fed *Faf2* knockdown mice, although the levels remained unchanged in livers (Figure 6D, E), while plasma β-hydroxybutyrate levels showed no significant change (Figure 6F). Our data suggest that FAF2 may regulate liver PCSK9 levels, potentially through the FOXO3-SIRT6 pathway, thereby contributing to the amelioration of ethanol-induced steatosis and the reduction in circulating LDL cholesterol.

### *Faf2* knockdown leads to an upregulation of ATGL activator, CGI-58 and downregulation of ATGL transport inhibitor, ELMOD2

**FIGURE 7:**
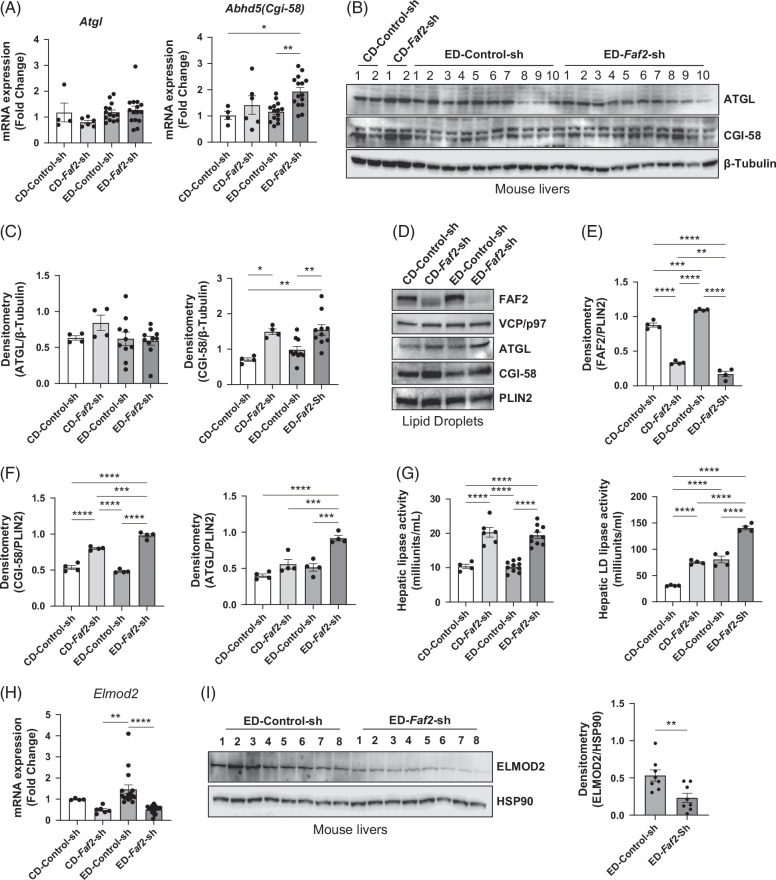
*Faf2* knockdown caused ATGL activation in the mouse liver. (A, B) Relative mRNA (A) and protein expression (B) of ATGL and CGI-58 in the livers of mice expressing either control shRNA or *Faf2*-shRNA, fed with either a control or ethanol diet. (C) Densitometric analysis of ATGL and CGI-58 protein levels. (D, E) Representative immunoblotting images (D) showing FAF2, VCP/p97, ATGL, CGI-58, and PLIN2 (loading control) in isolated lipid droplets, along with densitometric analysis (E) from mice expressing *Faf2*-shRNA or control shRNA and fed with control or ethanol diets. (G) Lipase activity was measured in the liver (left) and in isolated lipid droplets (right). (H, I) mRNA (H) and protein expression (I) of ELMOD2 in the livers of ethanol-fed mice expressing control shRNA or *Faf2*-shRNA, along with densitometric analysis of ELMOD2 protein levels (right). Data are presented as mean±SEM, with statistical significance indicated as *p<0.05; **p<0.01; ***p<0.001; ****p<0.0001 compared to the indicated group. Abbreviations: ATGL, adipose triglyceride lipase; CD, control diet; CGI-58, comparative gene identification-58; ED, ethanol-containing diet; ELMOD2, ELMO Domain Containing 2; FAF2, Fas-associated factor 2.

ATGL is a pivotal enzyme in lipolysis, catalyzing the initial breakdown of TGs into fatty acids. Liver-specific knockout or knockdown of ATGL can lead to hepatic steatosis.^32^,^33^ However, changes in ATGL activity may not always correlate with alterations in its mRNA or protein levels, as ATGL can be activated and translocated to LDs without an increase in its overall expression.^10^,^34^ In this study, we observed no significant differences in ATGL expression levels in liver tissues between the control and *Faf2* knockdown groups (Figure 7A, left, and Figure 7B, C, left). However, we found the ATGL levels increased in LDs isolated from *Faf2* knockdown livers compared to controls (Figure 7D–F). Comparative gene identification-58 (CGI-58), an activator of ATGL, can enhance ATGL’s TG hydrolase activity by approximately 20-fold, promoting the release of fatty acids from LDs.^35^,^36^,^37^ To further investigate the possible mechanism of FAF2-regulated ATGL activation and translocation, we detected the level of CGI-58 and found both the mRNA and protein were significantly upregulated in the *Faf2* knockdown livers (Figure 7A, right, and Figure 7B, C) and LDs (Figure 7D and F).

Since ATGL is the rate-limiting enzyme for lipolysis in LDs,^8^,^36^ the lipase activity could reflect ATGL enzyme activity. Therefore, we performed lipase activity assays in liver tissues of *Faf2* knockdown mice and found it was elevated regardless of dietary conditions (Figure 7G). In isolated LDs, lipase activity was also significantly increased in response to *Faf2* knockdown (Figure 7G, right). In addition, the assay’s sensitivity was validated using ATGL inhibitor treatment, as shown in Supplemental Figure S7C, http://links.lww.com/HC9/B886. This increase in lipase activity may be attributed to the activation of ATGL, mediated by elevated CGI-58 levels in the context of *Faf2* knockdown.

ELMOD2 is essential for regulating the transport of ATGL from the ER to LDs, thereby playing a critical role in cellular lipid metabolism.^10^ To explain how ATGL levels increased in LDs through the translocation, we explored our RNA sequencing data and found a significant downregulation of the Elmod2 levels in *Faf2* knockdown livers compared to controls (Figure 3D), which was further validated using qPCR and western blot analyses (Figure 7H, I). This reduction of ELMOD2 in *Faf2* knockdown livers may contribute to the increased levels of ATGL in LDs and translocation from the ER.

Our findings demonstrate that liver-specific *Faf2* knockdown induces lipase activity in both liver tissue and isolated LDs. This enhanced lipase activity could be attributed to ATGL activation through CGI-58 and its increased presence in LDs due to the downregulation of ELMOD2. Collectively, these results suggest that hepatic *Faf2* knockdown may ameliorate alcohol-induced steatosis by enhancing lipolytic activity through increased ATGL transport to LDs and its activation by CGI-58 and ELMOD2.

## DISCUSSION

ALD involves a complex cascade of events leading to various histopathological changes in the liver, with mechanisms underlying alcohol-induced liver injury remaining multifaceted.^38^ Previous genome-wide association study identified an association between FAF2 and alcohol-associated cirrhosis.^12^ However, its specific role in ALD has remained unclear.

**FIGURE 8:**
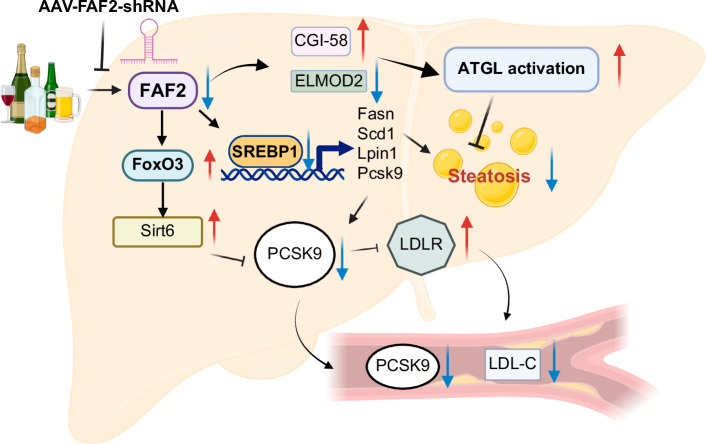
Proposed mechanisms of *Faf2* knockdown in lipid metabolism regulation in ALD. Knocking down alcohol-induced FAF2 by AAV-driven shRNA leads to several important changes in lipid metabolism. Specifically, *Faf2* knockdown increases the levels of CGI-58, a coactivator of ATGL, and inhibits ELMOD2, thereby promoting the transport and recruitment of ATGL to lipid droplets and enhancing the lipolytic activity to breakdown of triglycerides within these droplets. Additionally, modulation of SREBP1 or FOXO3-SIRT6 pathways and its downstream targets, including PCSK9 results in reduced hepatic steatosis and blood LDL levels. Abbreviations: AAV, adeno-associated virus; ATGL, adipose triglyceride lipase; FAF2, Fas-associated factor 2; LDL-C, low-density lipoprotein cholesterol; LDLR, low-density lipoprotein receptor; PCSK9, proprotein convertase subtilisin/kexin type 9; shRNA, short hairpin RNA; SREBP1, Sterol Regulatory Element Binding Protein 1.

In our study, we observed a significant increase in FAF2 expression in hepatocytes following alcohol exposure in both humans and mice. Suppression of FAF2 expression alleviated alcohol-induced liver steatosis through ATGL activation and SREBP1 pathway, as well as reducing plasma LDL levels via the PCSK9 pathway. Targeted liver-specific knockdown of *Faf2* in mice mitigated alcohol-induced steatosis. Comparative gene expression analysis between ethanol-fed control and *Faf2* knockdown mouse livers identified differential expression of multiple lipid metabolism genes regulated by SREBP1. Notably, PCSK9 was downregulated upon *Faf2* knockdown, which may have contributed to the reduction in blood LDL levels through regulation by either the SREBP1 or FOXO3-SIRT6 pathway. Additionally, we found that *Faf2* knockdown enhanced ATGL lipolytic activity in the liver, potentially due to increased levels of CGI-58 (an ATGL coactivator) or through reduced expression of ELMOD2, which is involved in ATGL transport to LDs (Figure 8). These findings underscore the critical role of FAF2 in alcohol-induced hepatic steatosis and suggest a mechanism for FAF2 involvement in PCSK9-mediated regulation of circulating LDL-C levels.

To investigate the molecular mechanisms underlying the effect of *Faf2* knockdown on alcohol-induced liver steatosis, we performed RNA sequencing and bioinformatic analysis. Our data revealed differential expression of genes enriched in several lipid metabolism pathways, including fatty acid degradation, linoleic acid metabolism, and the biosynthesis of unsaturated fatty acids (Figure 3). Additionally, pathways related to the positive regulation of fatty acid biosynthesis, TG biosynthesis, and LD organization were also significantly enriched (Supplemental Figures S5 and S6, http://links.lww.com/HC9/B886). These findings suggest that FAF2 may exert its effects primarily through the regulation of pathways associated with hepatic steatosis.

Further investigation into transcription factor regulation of these DEGs identified SREBP1 as a key regulator. Recent studies have indicated that *Faf2* knockdown can attenuate SREBP1 cleavage, thereby influencing lipid metabolism.^39^ In our study, we observed reductions in both mRNA and precursor forms of SREBP1 following *Faf2* knockdown, although cleaved SREBP1 levels could not be conclusively determined due to low detection of cleaved SREBP1 bands. This reduction in SREBP1 and its downstream target genes likely contributes to the protective effect of *Faf2* knockdown against alcohol-induced steatosis.

Among the genes significantly altered in our study, PCSK9 emerged as an intriguing candidate. To date, no established connection exists between FAF2 and PCSK9 in the regulation of steatosis in ALD. PCSK9 is primarily expressed in the liver, where it plays a crucial role in controlling LDL-C levels by interacting with the LDLR.^29^,^40^ In circulation, PCSK9 binds to LDLRs on hepatocyte surfaces, promoting their degradation. Loss-of-function mutations in PCSK9 are associated with decreased LDL-C levels and a reduced risk of cardiovascular disease.^41^,^42^ Clinical trials and animal studies using PCSK9 inhibitors have suggested potential benefits in reducing liver steatosis, inflammation, fibrosis, and cardiovascular risk.^43^,^44^ Our study demonstrated that Faf2 knockdown significantly reduced PCSK9 expression at both mRNA and protein levels, indicating that FAF2 may regulate PCSK9 expression and, thus, play a critical role in the development of liver steatosis. Interestingly, PCSK9 has been identified as a target gene of SREBP1.^45^ Furthermore, Tao et al^31^ reported that FOXO3 and SIRT6 could regulate PCSK9 in the liver, with SIRT6 recruited by FOXO3 to deacetylate histone H3 at lysines 9 and 56 on the *Pcsk9* promoter, leading to its repression. In support of this, our data revealed significant upregulation of FOXO3 and SIRT6 in *Faf2* knockdown mouse livers, suggesting an additional mechanism by which FAF2 may modulate PCSK9 levels.

Given FAF2’s documented role in modulating ATGL activity and its involvement in the ATGL-mediated lipolysis pathway,^13^ we aimed to explore its regulation under ALD conditions. Previous studies have shown that ATGL activity can be influenced by coactivators, inhibitors, or transporters, often without affecting ATGL mRNA or protein levels.^10^,^46^ For instance, CGI-58 is known to directly activate ATGL through binding.^36^ In our study, we observed a significant increase in CGI-58 transcript levels in *Faf2* knockdown livers, indicating a possible enhancement of ATGL activity when FAF2 is reduced. Supporting this, we found elevated lipase activity in both whole liver lysates and isolated LDs from Faf2-knockdown livers.

LDs serve as the primary storage site for TGs, with ATGL acting as the rate-limiting enzyme in TG breakdown, converting them into free fatty acids and diacylglycerol.^47^ Thus, activating ATGL through FAF2 reduction in ALD-related steatosis may offer a promising therapeutic strategy. Moreover, ELMOD2 inhibits adenosine diphosphate-ribosylation factor 1-coat protein complex 1 activity, which reduces ATGL recruitment to LDs, leading to increased TG accumulation in vitro.^10^ Consistent with these findings, our data showed that Faf2 knockdown decreased both transcript and protein levels of ELMOD2 (Figures 3 and 7). Lower ELMOD2 levels may facilitate greater recruitment of ATGL to LDs, thereby enhancing TG breakdown and reducing hepatic steatosis through increased ATGL activity.

Our findings underscore the critical role of FAF2 in ALD, though certain limitations remain. First, we did not explore the mechanism by which alcohol elevates FAF2 expression, as transcriptional regulation of the FAF2 promoter is yet to be investigated. Second, our use of a shRNA vector with a pol III promoter was not cell specific; however, the AAV8 vector and tail vein injection likely enriched the knockdown effect in the liver. Future studies using tissue-specific or cell-specific knockout models would allow us to pinpoint the specific cell types, contributing to the protective effects of FAF2 knockdown in ALD. Lastly, we did not evaluate fibrosis in this study, as our model does not readily develop this pathology.

In summary, this study suggests that FAF2 knockdown may alleviate alcohol-induced liver steatosis through 2 primary mechanisms: (1) modulation of SREBP1 and its downstream targets, including PCSK9, and (2) enhancement of ATGL-mediated lipolysis. Future research using additional animal models and FAF2-specific knockout mice is warranted to further elucidate the pathways by which FAF2 influences liver steatosis, supporting its potential as a therapeutic target for ALD.

## Supplementary Material

## Data Availability

The raw RNA-﻿sequencing data were deposited in the NCBI Gene Expression Omnibus under accession number GSE270659. All data, analytic methods, and study materials will be made available to other researchers upon request. Nazmul Huda, Suthat Liangpunsakul, and Zhihong Yang: study concept and design; Nazmul Huda and Zhihong Yang: acquisition of data, analysis, and interpretation of data; Jing Ma, Praveen Kusumanchi, Hui Gao, Themis Thoudam, Ge Zeng, and Yanchao Jiang: critical revision of the manuscript; Nicholas J. Skill and Zhaoli Sun: providing human liver samples, Nazmul Huda, Zhihong Yang, and Suthat Liangpunsakul: drafting and finalizing the manuscript. All authors have read and approved the manuscript for submission.
