# Supplementary material for: Silencing FAF2 mitigates alcohol-induced hepatic steatosis by modulating lipolysis and PCSK9 pathway
Source: Hepatol Commun. 2025 Feb 19;9(3):e0641. doi: 10.1097/HC9.0000000000000641 (PMC11841855; doi:10.1097/HC9.0000000000000641)
Supplement: Supplementary file 1 [file hc9-9-e0641-s001.docx]

**Table 1: Nucleotide sequences of *Faf2*-shRNA**

**Table 2: qPCR primers used in this study**

| **Origin** | **Gene Name** | **Forward Primer** | **Reverse Primer** |
| --- | --- | --- | --- |
| Mouse | *Faf2* | GAGCAGGATCTAACTCAGGAGC | CAGCAGCCTCCATGTTCCAG |
| Human | *Faf2* | AGCGGGATCTAACCCAGGAG | GTTCCAAGGTATGGCGACACT |
| Mouse | *Pcsk9* | GAGACCCAGAGGCTACAGATT | AATGTACTCCACATGGGGCAA |
| Mouse | *Foxo3* | CTGGGGGAACCTGTCCTATG | TCATTCTGAACGCGCATGAAG |
| Mouse | *Sirt6* | ATGTCGGTGAATTATGCAGCA | GCTGGAGGACTGCCACATTA |
| Mouse | *Atgl(Pnpla2)* | CGCCTCTCGAAGGCTCTCT | TGTAGCCCTGTTTGCACATCTC |
| Mouse | *Abhd5(Cgi-58)* | GTGAGACAGCTTTCAAAAACATGAC | CACCTATCCGCTGAAGCATTG |
| Mouse | *Elmod2* | TGGACACTTTTTCCGATTTTGGA | ACTCGTGTTGCATTCTGTAGGA |
| Mouse | *Aldh1* | GCAAAGCTGCGGTGCTATG | TCACACAAGTCACCCCTTCTC |
| Mouse | *Cyp2E1* | CGTTGCCTTGCTTGTCTGGA | AAGAAAGGAATTGGGAAAGGTCC |
| Mouse | *Tnfα* | AGGCTGCCCCGACTACGT | GACTTTCTCCTGGTATGAGATAGCAAA |
| Mouse | *IL-1β* | TCGCTCAGGGTCACAAGAAA | CATCAGAGGCAAGGAGGAAAAC |
| Mouse | *IL-6* | TCCATCCAGTTGCCTTCTTG | TTCCACGATTTCCCAGAGAAC |
| Mouse | *F4/80* | CTTTGGCTATGGGCTTCCAGTC | GCAAGGAGGACAGAGTTTATCGTG |
| Mouse | *Ly6g* | TGCGTTGCTCTGGAGATAGA | CAGAGTAGTGGGGCAGATGG |
| Mouse | *Icam1* | CAATTTCTCATGCCGCACAG | AGCTGGAAGATCGAAAGTCCG |
| Mouse | *Vcam1* | TGAACCCAAACAGAGGCAGAGT | GGTATCCCATCACTTGAGCAGG |
| Mouse | *Nlrp3* | AAGTAAGGCCGGAATTCACC | AAAATGCCTTGGGAGACTCA |
| Mouse | *Ccl2* | ATTGGGATCATCTTGCTGGT | CCTGCTGTTCACAGTTGCC |
| Mouse | *Ccl3* | GTGGAATCTTCCGGCTGTAG | ACCATGACACTCTGCAACCA |
| Mouse | *Ccl4* | GAAACAGCAGGAAGTGGGAG | CATGAAGCTCTGCGTGTCTG |
| Mouse | *Cxcl1* | ACTGCACCCAAACCGAAGTC | TGGGGACACCTTTTAGCATCTT |

**Table 3: Antibodies used in this study**

| **Antibody/host** | **Source/catalogue number/dilution** |
| --- | --- |
| FAF2/Rabbit | Genetex/GTX115680/WB: 1:1000; IF: 1:400/ IHC:1:200 |
| GAPDH/Rabbit | Sigma-Aldrich/SAB2108266/WB:1:5000 |
| FOXO3/Rabbit | Sigma-Aldrich/SAB3500508/WB:1:1000 |
| SIRT6/Rabbit | Sigma-Aldrich/S4322/WB:1:1000 |
| PCSK9/Goat | Sigma-Aldrich/SAB2501521/WB:1:1000 |
| LDLR/Rabbit | Cayman/10007665/WB:1:1000 |
| ELMOD2/Rabbit | ProteinTech/13027-1-1AP/WB: 1:1000 |
| HNF4α/Rabbit | Sigma-Aldrich/ZRB1457/IF 1:1000, WB; 1:2000 |
| HNF4α/Mouse | Perseus Proteomics/PP-K9218-00/IF: 1:100 |
| HSP90/mouse | Abcam/ab13492/WB:1:1000 |
| MPO/Rabbit | Biocare/PP023AA/pre-diluted |
| F4/80/Rabbit | Cell Signaling Tech/ 70076S/1:400 |
| 4-HNE/Mouse | Jaica/HNEJ-2/1:400 |
| PDI/Rabbit | Cell Signaling Tech/ 2446/IF:1:50 |
| TOM20/Rabbit | Cell Signaling Tech/ 42406/IF:1:200 |
| Rab7/Rabbit | Cell Signaling Tech/ 9367/IF:1:50 |
| LAMP2/Rabbit | Cell Signaling Tech/ 34141/IF:1:1,500 |
| FLAG/Mouse | Sigma-Aldrich/1804/1:200 |
| Alexa Fluor 488/Rabbit | Invitrogen/A11008/1:200 |
| Alexa Fluor 568/Mouse | Invitrogen/A11004/1:200 |
| HRP-anti-Mouse/Goat | Cell Signaling Tech/ 8125S/pre-diluted for IHC |
| HRP-anti-Rabbit/Goat | Cell Signaling Tech/ 8114S/pre-diluted for IHC |
| HRP-anti-Rb IgG/Goat | Cell Signaling Tech/ 7074S/1:3000 |
| HRP-anti-Ms IgG/Goat | Cell Signaling Tech/ 7076S/1:3000 |
| Cy™3 AffiniPure Donkey Anti-Rabbit IgG | Jackson ImmunoRes/711-165-152/1:200 |
| Cy™2 AffiniPure Donkey Anti-Rabbit IgG | Jackson ImmunoRes/711-225-152/1:200 |
| Cy™3 AffiniPure Donkey Anti-mouse IgG | Jackson ImmunoRes/711-165-150/1:200 |
| Cy™2 AffiniPure Donkey Anti-mouse IgG | Jackson ImmunoRes/ 715-225-150/1:200 |
