# Supplementary material for: Silencing FAF2 mitigates alcohol-induced hepatic steatosis by modulating lipolysis and PCSK9 pathway
Source: Hepatol Commun. 2025 Feb 19;9(3):e0641. doi: 10.1097/HC9.0000000000000641 (PMC11841855; doi:10.1097/HC9.0000000000000641)
Supplement: Supplementary file 2 [file hc9-9-e0641-s002.docx]

**SUPPLEMTARY MATERIALS**

**Silencing FAF2 Mitigates Alcohol-induced Hepatic Steatosis by Modulating Lipolysis and PCSK9 Pathway**

*Short Title***: Silencing FAF2 ameliorates hepatic steatosis**

Nazmul Huda^1^, Praveen Kusumanchi^1^, Yanchao Jiang^1^, Hui Gao^1^, Themis Thoudam^1^, Ge Zeng^1,2^, Nicholas J Skill^3^, Zhaoli Sun^4^, Suthat Liangpunsakul^1,5,6^, Jing Ma^1#^, Zhihong Yang^1#^

^1^Division of Gastroenterology and Hepatology, Department of Medicine, Indiana University School of Medicine, Indianapolis, IN, ^2^Department of Infectious Diseases, Nanfang Hospital, Southern Medical University, Guangzhou, China; ^3^Louisiana State University Health Science Center, New Orleans, Louisiana, USA, ^4^Department of Surgery, John Hopkins University, Baltimore, MD, ^5^Department of Biochemistry and Molecular Biology, Indiana University School of Medicine, Indianapolis, IN, ^6^Roudebush Veterans Administration Medical Center, Indianapolis, IN.

**Supplementary methods:**

**Cell culture and *in vitro* ethanol treatment**

The VL-17A cell line, gifted from Dr. Dahn L. Clemens at the University of Nebraska Medical Center, and AML-12 cells from the American Type Culture Collection (ATCC) were utilized in this study. Both cell lines were cultured in DMEM (Gibco, #11965092, Waltham, MA) supplemented with 10% fetal bovine serum (Corning® Fetal Bovine Serum, Woodland, CA), 1% antibiotics (Anti-Anti, Gibco), and 2 mM glutamine (Gibco GlutaMAX). Cultures were maintained in a humidified atmosphere at 37°C with 5% CO2. The cells were transfected with *Flag-Faf2* or infected with pAAV-*ZsGreen-Faf2*-shRNA for 24 hours, then treated with 75 mM ethanol for another 24 hours before being harvested for immunofluorescence staining.

**Quantitative real-time PCR (qPCR) analysis**

In this study, total RNA from liver tissue or cell lines was extracted using Trizol reagent (Invitrogen, Carlsbad, CA) or the RNeasy Plus Micro Kit (Qiagen, Hilden, Germany). Subsequently, cDNA was synthesized from 2 µg of RNA using a high-fidelity cDNA synthesis kit (Thermo Fisher Scientific, Waltham, MA) through a reverse transcriptase reaction. The resulting cDNA was diluted at a 1:5 ratio with nuclease-free water prior to use. For quantitative real-time PCR (qPCR) analyses, 1 µL of the diluted cDNA was mixed with iTaq Universal SYBR Green Mix (Bio-Rad, Hercules, CA) and amplified using either the CFX 384 or CFX96 Real-Time System (Bio-Rad, Hercules, CA). The primer sequences used for qPCR are listed in **Supplementary Table 2**.

**Immunoblotting**

Immunoblotting was performed following standard protocols. Primary antibodies specific to target proteins (listed in **Supplementary Table 3**) were incubated with the membranes overnight at 4°C. Imaging and quantification of protein bands were carried out using a ChemiDoc Imaging System (Bio-Rad, Hercules, CA). Densitometric analysis was conducted using image analysis software (ImageJ, version 1.54) to quantify relative protein expression levels.

**Mouse primary hepatocyte isolation**

The detailed protocol for hepatocyte isolation is referenced elsewhere(25). In summary, after inducing anesthesia with isoflurane, the livers from both control and ethanol diet fed mice were perfused with an EDTA-containing buffer to disrupt cell-cell junctions. Collagenase (Sigma-Aldrich, St. Louis, MO) was then used to enzymatically digest the extracellular matrix. The resulting liver tissue suspension was filtered through a 70 µm cell strainer into a 50 mL tube and centrifuged at 50 × g for 5 minutes at 4°C to pelletize the hepatocytes. The supernatant, containing nonparenchymal cells (NPC), was collected. The hepatocyte pellet was resuspended in 10 mL of William's Medium E and layered onto a freshly prepared mixture of Percoll and William's Medium E (1:1) in a 20 mL volume. This suspension was then centrifuged at 1500 × g for 8 minutes. Viable hepatocytes were carefully collected from the interface, counted, and plated on collagen-coated plates for subsequent experiments.

**Biochemical analysis**

Plasma levels of ALT, AST, TG, total cholesterol, LDL-cholesterol, and free fatty acids (FFA) were quantified using specific colorimetric assays with the following kits: ALT (Cat # A524, TECO Diagnostic, Anaheim, CA), AST (Cat # A7561, Pointe Scientific, Skokie, IL), TG (Cat # T7532, Pointe Scientific, Skokie, IL), total cholesterol (Cat # C7510, Pointe Scientific, Skokie, IL), LDL-cholesterol (Cat # 79980, Crystal Chem, IL), and FFA (Cat # MAK044, Sigma-Aldrich, St. Louis, MO). Plasma levels of PCSK9 were determined using an ELISA kit (MPC900, R&D Systems, MN, USA). Hepatic lipase activity was measured using the Lipase Activity Assay Kit II (Cat # MAK047, Sigma, MO, USA) in both liver tissue and isolated lipid droplets, following the manufacturer's instructions. Briefly, liver tissue lysates were prepared using the lysis buffer provided with the kit. The lysates were either untreated or treated with 40 µM Atglistatin (an ATGL inhibitor) for 1 hour at 37°C prior to conducting the lipase activity assay(25-27). Mouse plasma β-hydroxybutyrate levels were analyzed by ELISA using a commercially available kit (catalog #700190; Cayman Chemicals, Ann Arbor, MI). For hepatic triglyceride (TG) and total cholesterol measurements, a modified protocol based on Zou et al. (28) was employed. Briefly, 50 mg of liver tissue was homogenized in 300 µL of chloroform (1:2). An additional 300 µL of chloroform was added, and the mixture was homogenized again. The lipid layer obtained after centrifugation at 800 × g for 10 minutes was transferred to a fresh tube. After air-drying, the lipids were suspended in 5% Triton X-100, and hepatic TG and cholesterol levels in the lipid extract were determined using the aforementioned kits.

**Supplementary Figure legends:**

**Supplementary Figure 1**: Ethanol stimulated upregulation of FAF2 expression in the liver. (A) Representative images of immunohistochemistry (IHC) analysis of FAF2 protein expression in pair-fed and ethanol-fed mice liver (B, C) Relative mRNA expression of *Faf2* from Hepatocytes isolated from mice (regular diet) liver and treated with ethanol in vitro. (C) Representative western blot (left panel) and densitometric analysis of FAF2 protein in isolated Hepatocytes from mice (regular diet) liver and treated with ethanol in vitro. Right panel shows a densitometric analysis. Scale bar: 25 µm for top two panels, and 8 µm for bottom panel. (D, E) Immunofluorescence and Western blot and analysis of FAF2 protein expression in ethanol treated AML12 cell, respectively. (F) Western blot (left panel) and densitometric analysis of FAF2 protein in VL-17A cell line treated with ethanol. (G) Immunofluorescence images stained with FAF2 antibody (red), and BODIPY (lipid droplets, green) in ethanol treated VL-17A. Scale bar: 25 µm. (C) (D) (F) & (G) Data are presented as mean ± SEM, **P<0.01; ***P<0.001; ****P<0.0001 vs indicated group.

**Supplementary Figure 2**: Subcellular location of FAF2 in AML12 cell line. (A-C) AML2 cells were transfected with FLAG-FAF2 for 24 hours before treated with 75 mM EtOH for another 24 hours, followed by immunofluorescence staining. FAF2 was stained with FLAG antibody (red). The cells were co-stained with PDI (ER marker, green in A), RAB7 (endosome marker, green in B) or LAMP2 (lysosome marker, green in C), separately. Scale bar: 8 µm.

**Supplementary Figure 3**: Evaluation of FAF2-ShRNAs in AML12 cell line. (A) Relative mRNA expression of *Faf2* in AML12 cell line transfected with 3 different shRNAs. (B) Western blot images showing the level of knock down of FAF2 protein via 3 different shRNAs in AML12 cell line. (C) Immunofluorescence of FAF2 in AML12 harboring plasmids containing either control shRNA and ZS-Green1gene (Top panel) or Faf2-shRNA #3 (Supplementary Table 1) and ZS-Green1gene (bottom panel). Scale bar: 25 µm. (D) Relative mRNA expression of Faf2 (C) in adipose tissues from control (CD) or ethanol diet (ED) group harboring either control-shRNA or Faf2-shRNA. Data are presented as mean ± SEM, **P<0.01; ***P<0.001; ****P<0.0001 vs indicated group.

**Supplementary Figure 4**: Analysis of inflammatory markers in FAF2-KD liver. (A-C) mRNA expression analysis of inflammation associated genes (*Tnfα, Il-1b, Il6, F4/80, Ly6g, Icam1, Vcam1, Nlrp3, Ccl2, Ccl3, Ccl4, & Cxcl1*) in the livers expressing either control shRNA or Faf2-ShRNA in mice fed with either control or ethanol-diet. (D) Representative immunohistochemistry image and quantitative analysis of MPO and F4/80 staining in the liver sections harboring either control shRNA or Faf2-ShRNA in mice fed with either control or ethanol-diet. Scale bar: 200 µm. (E) Liver total cholesterol in the mice expressing either control-shRNA or Faf2-shRNA fed with either control or ethanol-diet, respectively. Each dots representing an individual mouse. Data are presented as mean ± SEM, *P<0.05 vs indicated group.

**Supplementary Figure 5:** RNA-sequencing data suggest FAF2 regulates lipid metabolism in liver. (A) Heat map analysis of top 10 DEGs in retinol metabolism and linoleic acid metabolism pathways. (B) Heat map of top DEGs in AMPK signaling pathway. (C) Gene Ontology (GO) enrichment analysis of the DEGs in Fig. 3A.

**Supplementary Figure 6**: GSEA analysis of the RNA-sequencing data. (A) Dot plot of GSEA analysis using GO term. (B-C) Enrichment plots of indicated pathways.

**Supplementary Figure 7**: FAF2 regulated SREBP1 and its target genes. (A-B) Western blot detected SREBP1, FASN, LPIN protein levels with addition samples. Each line representing an individual mouse liver. The densitometric analysis was provided and ACTIN (beta-ACTIN) or GAPDH was used for loading control. Sample in arrow is the same sample, which was loaded in both gels. (C) Lipase activity assay after treated with ATGL inhibitor. Data are presented as mean ± SEM, *P<0.05; **P<0.01; ****P<0.0001 vs indicated group.
