# Supplementary material for: Silencing FAF2 mitigates alcohol-induced hepatic steatosis by modulating lipolysis and PCSK9 pathway
Source: Hepatol Commun. 2025 Feb 19;9(3):e0641. doi: 10.1097/HC9.0000000000000641 (PMC11841855; doi:10.1097/HC9.0000000000000641)

Supplementary Figure legends:

Supplementary Figure 1: Ethanol stimulated upregulation of FAF2 expression in the liver. (A) Representative images of immunohistochemistry (IHC) analysis of FAF2 protein expression in pair-fed and ethanol-fed mice liver (B, C) Relative mRNA expression of *Faf2* from Hepatocytes isolated from mice (regular diet) liver and treated with ethanol in vitro. (C) Representative western blot (left panel) and densitometric analysis of FAF2 protein in isolated Hepatocytes from mice (regular diet) liver and treated with ethanol in vitro. Right panel shows a densitometric analysis. Scale bar: 25 μm for top two panels, and 8 μm for bottom panel. (D, E) Immunofluorescence and Western blot and analysis of FAF2 protein expression in ethanol treated AML12 cell, respectively. (F) Western blot (left panel) and densitometric analysis of FAF2 protein in VL-17A cell line treated with ethanol. (G) Immunofluorescence images stained with FAF2 antibody (red), and BODIPY (lipid droplets, green) in ethanol treated VL-17A. Scale bar: 25 μm. (C) (D) (F) & (G) Data are presented as mean ± SEM, **P<0.01; ***P<0.001; ****P<0.0001 vs indicated group.

0

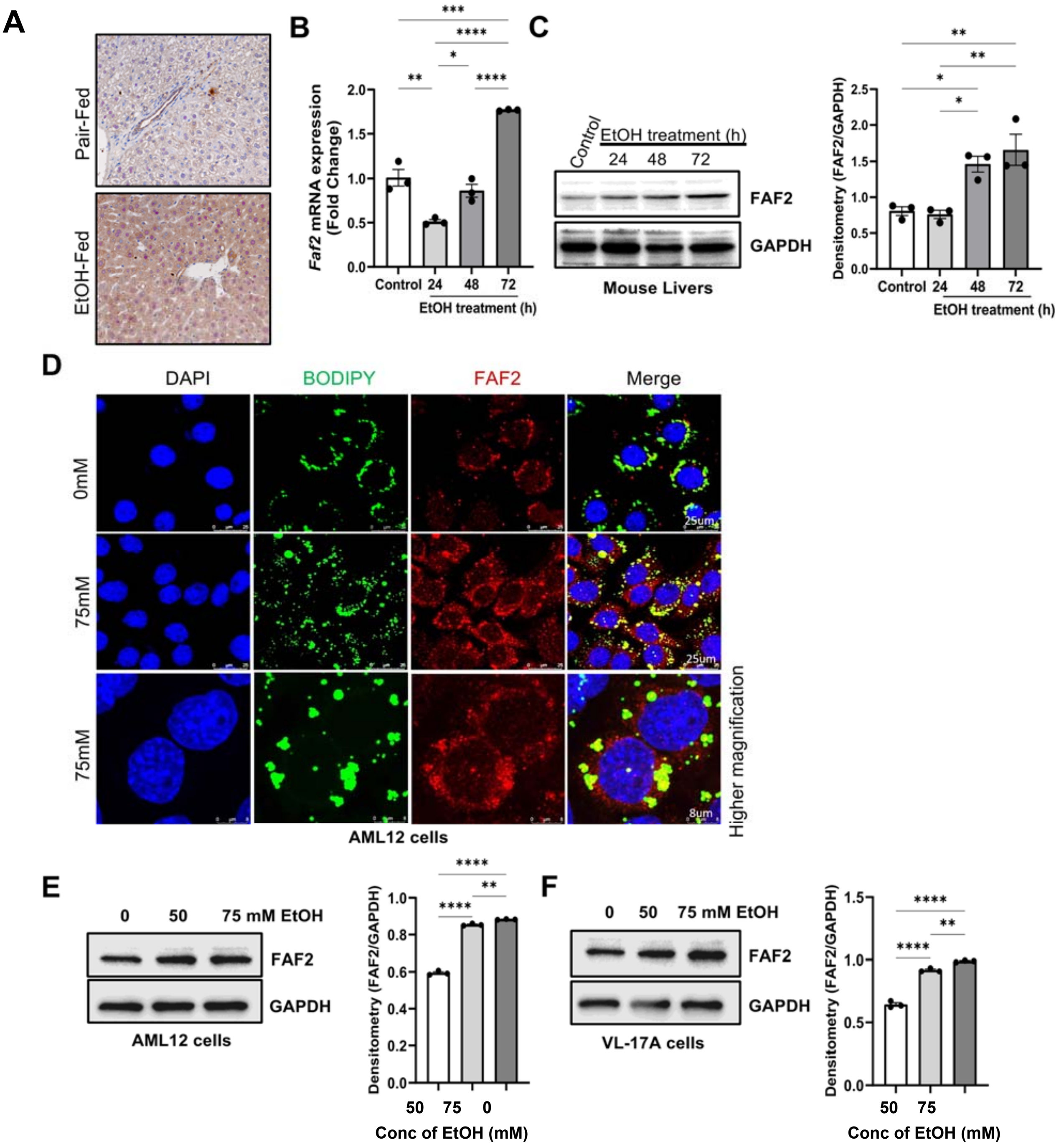

G

DAPI BODIPY FAF2 Merge

Supplementary Figure 2: Subcellular location of FAF2 in AML12 cell line. (A-C) AML2 cells were transfected with FLAG-FAF2 for 24 hours before treated with 75 mM EtOH for another 24 hours, followed by immunofluorescence staining. FAF2 was stained with FLAG antibody (red). The cells were co-stained with PDI (ER marker, green in A), RAB7 (endosome marker, green in B) or LAMP2 (lysosome marker, green in C), separately. Scale bar: 8 μm.

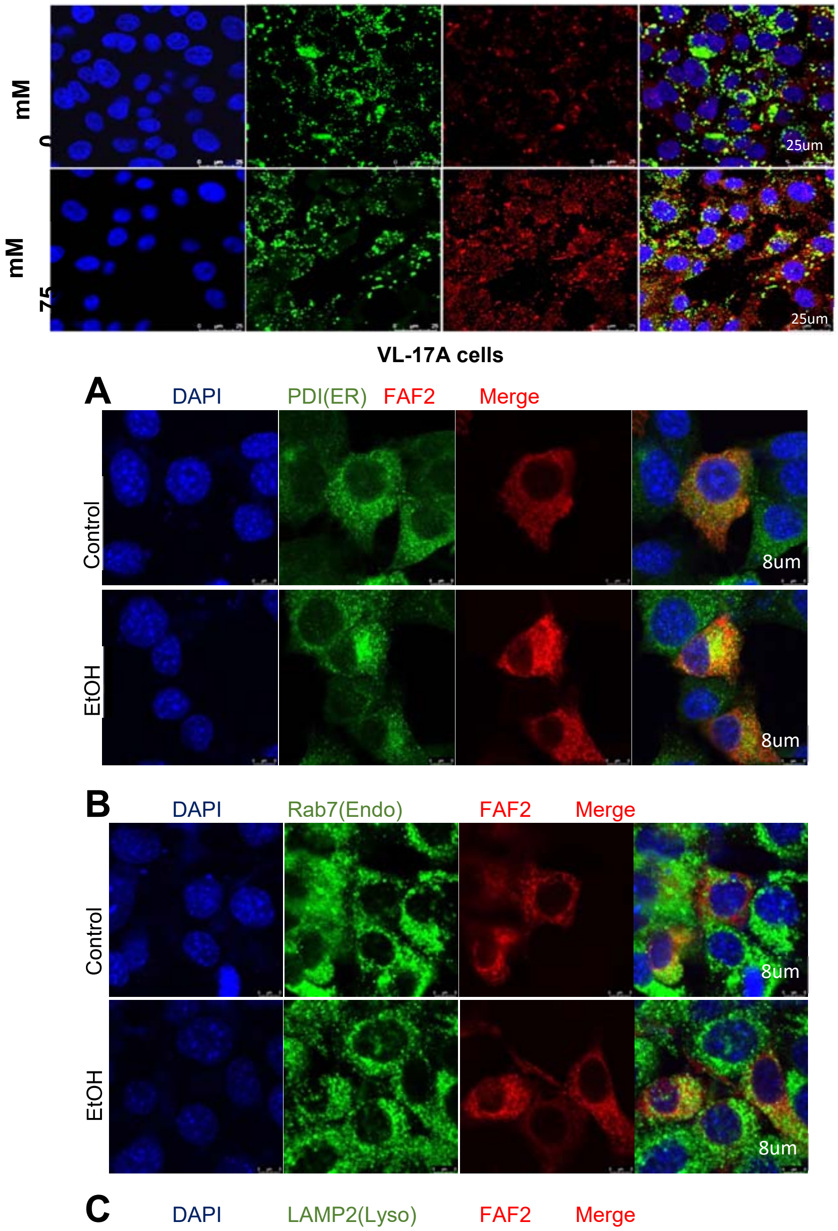

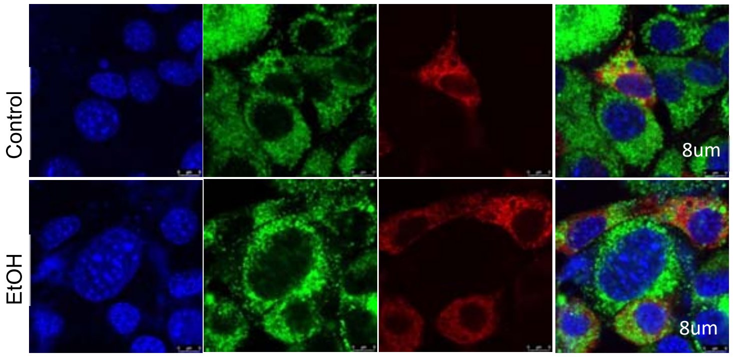

AML12 cells

Supplementary Figure 3: Evaluation of FAF2-ShRNAs in AML12 cell line. (A) Relative mRNA expression of *Faf2* in AML12 cell line transfected with 3 different shRNAs. (B) Western blot images showing the level of knock down of FAF2 protein via 3 different shRNAs in AML12 cell line. (C) Immunofluorescence of FAF2 in AML12 harboring plasmids containing either control shRNA and ZS-Green1gene (Top panel) or Faf2-shRNA #3 (Supplementary Table 1) and ZS-Green1gene (bottom panel). Scale bar: 25 µm. (D) Relative mRNA expression of Faf2 (C) in adipose tissues from control (CD) or ethanol diet (ED) group harboring either control-shRNA or Faf2-shRNA. Data are presented as mean ± SEM, **P<0.01; ***P<0.001; ****P<0.0001 vs indicated group.

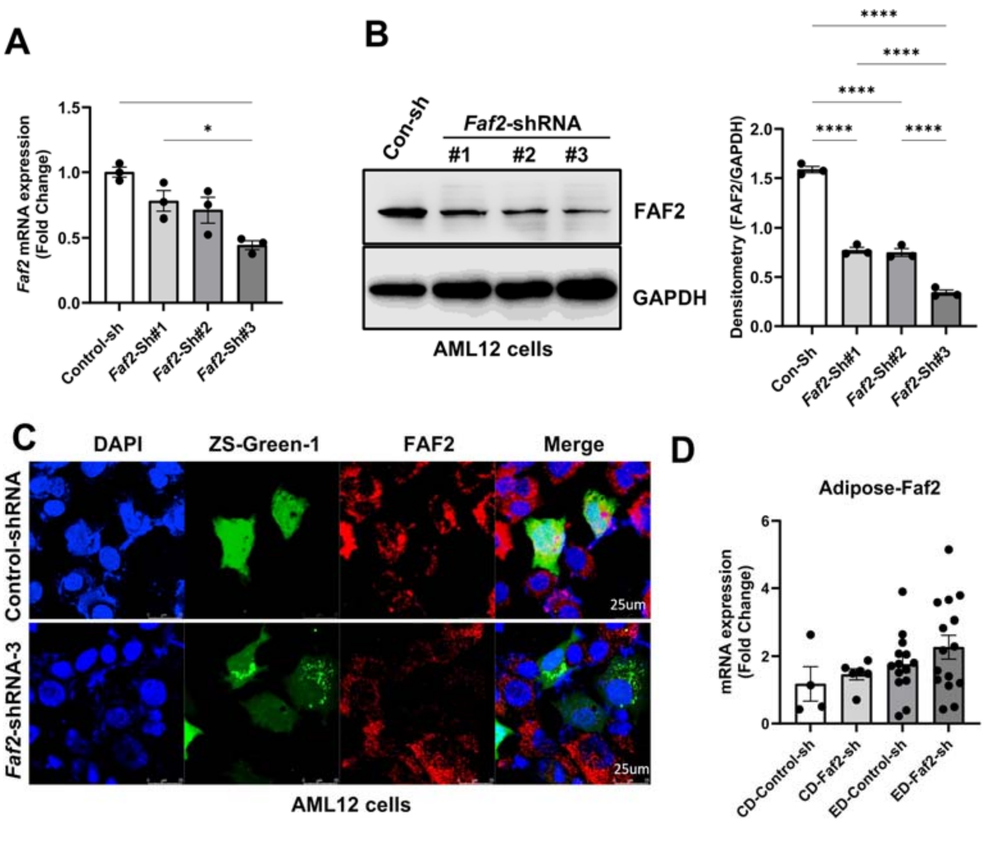

Supplementary Figure 4: Analysis of inflammatory markers in FAF2-KD liver. (A-C) mRNA expression analysis of inflammation associated genes (*Tnfα, Il-1b, Il6, F4/80, Ly6g, Icam1, Vcam1, Nlrp3, Ccl2, Ccl3, Ccl4, & Cxcl1*) in the livers expressing either control shRNA or Faf2-ShRNA in mice fed with either control or ethanol-diet. (D) Representative immunohistochemistry image and quantitative analysis of MPO and F4/80 staining in the liver sections harboring either control shRNA or Faf2-ShRNA in mice fed with either control or ethanol-diet. Scale bar: 200 μm. (E) Liver total cholesterol in the mice expressing either control-shRNA or Faf2-shRNA fed with either control or ethanol-diet, respectively. Each dots representing an individual mouse. Data are presented as mean ± SEM, *P<0.05 vs indicated group.

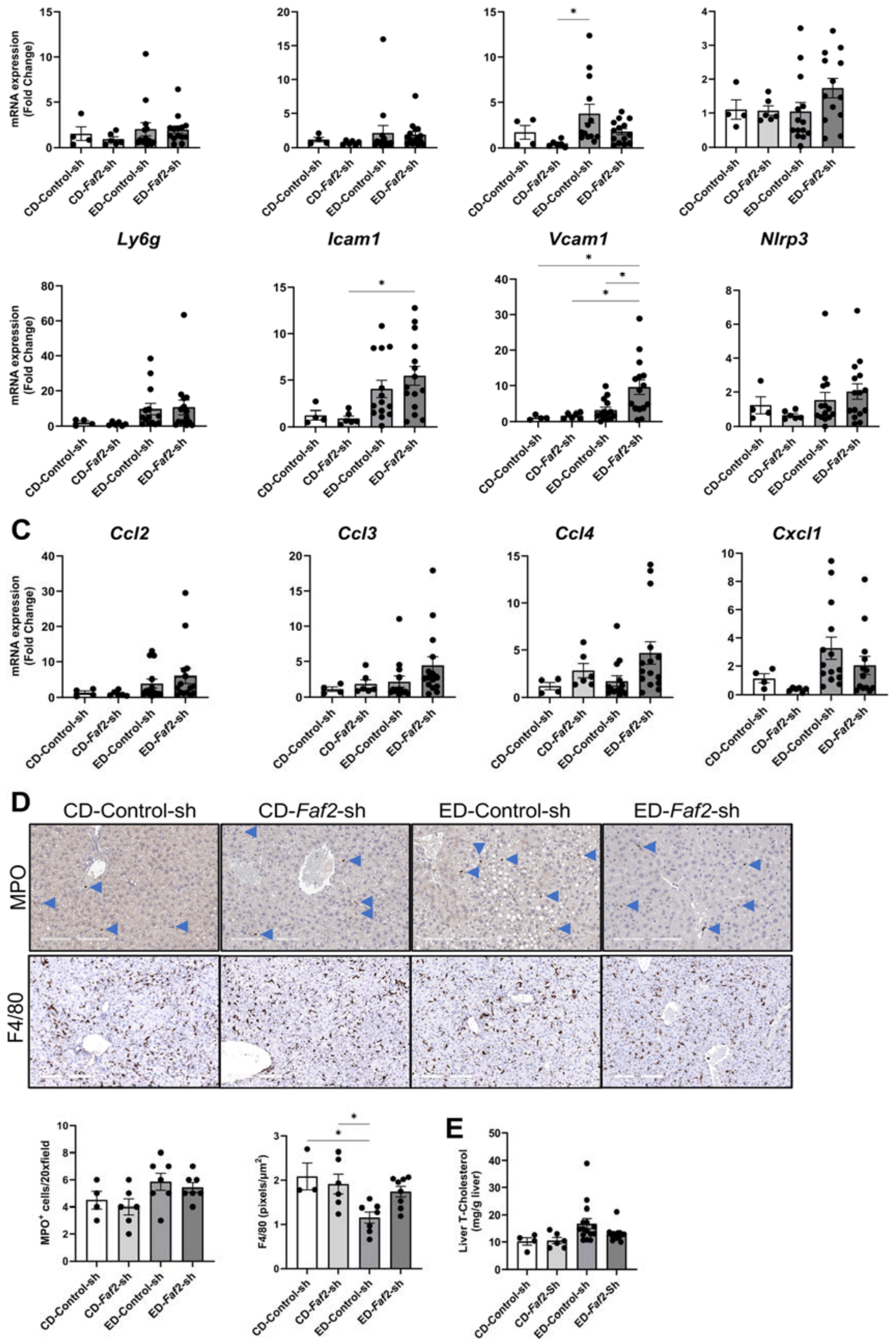

mRNA expression (Fold Change)
CD-Control-sh
CD-Faf2-sh
ED-Control-sh
ED-Faf2-sh
Ly6g
Icam1
Vcam1
Nlrp3
C
Ccl2
Ccl3
Ccl4
Cxcl1
D
MPO
F4/80
MPO+ cells/20xfield
F4/80 (pixels/µm2)
E
Liver T-Cholesterol (mg/g liver)
CD-Faf2-Sh
ED-Faf2-Sh

Supplementary Figure 5: RNA-sequencing data suggest FAF2 regulates lipid metabolism in liver. (A) Heat map analysis of top 10 DEGs in retinol metabolism and linoleic acid metabolism pathways. (B) Heat map of top DEGs in AMPK signaling pathway. (C) Gene Ontology (GO) enrichment analysis of the DEGs in Fig. 3A.

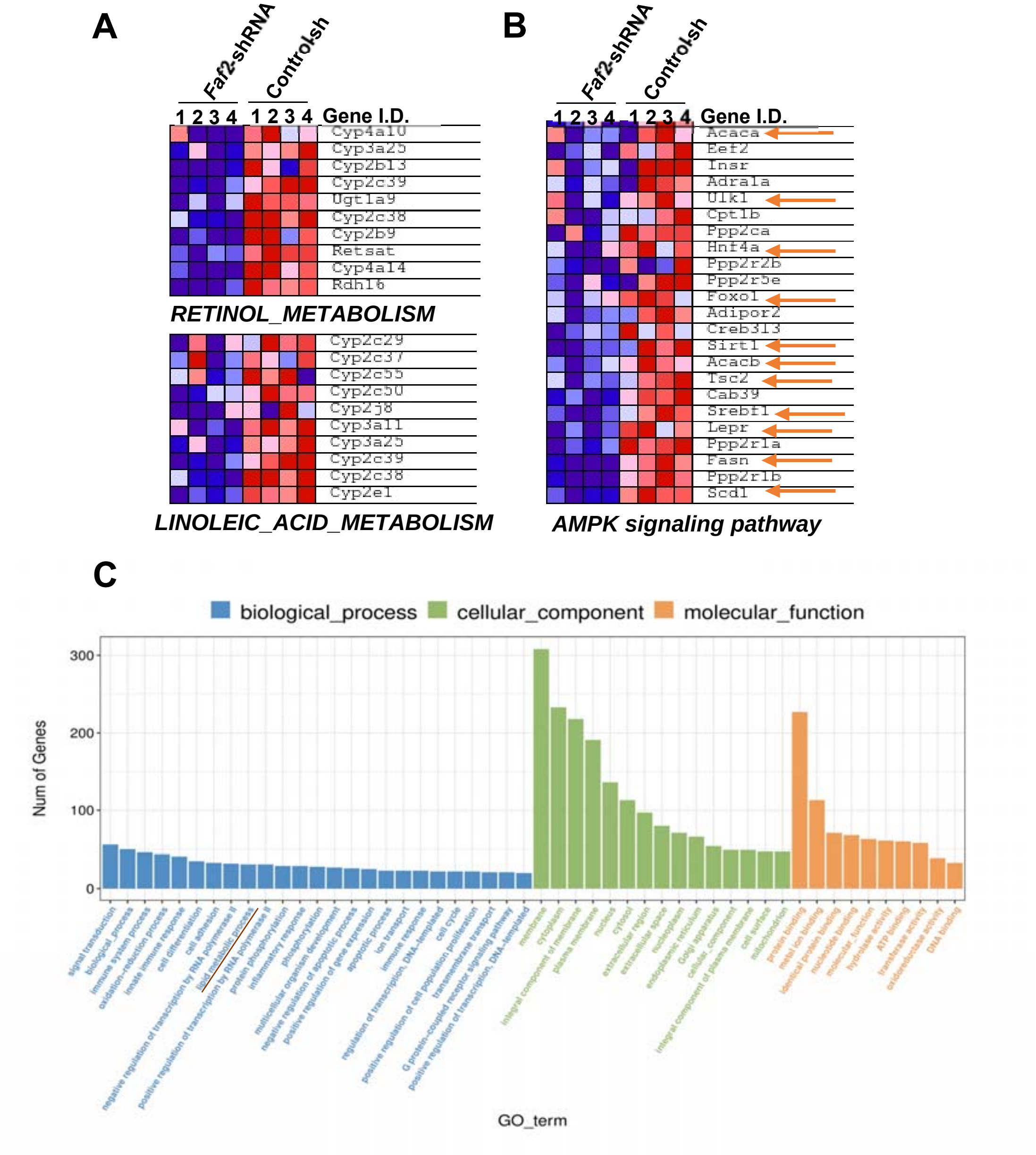

Supplementary Figure 6: GSEA analysis of the RNA-sequencing data. (A) Dot plot of GSEA analysis using GO term. (B-C) Enrichment plots of indicated pathways.

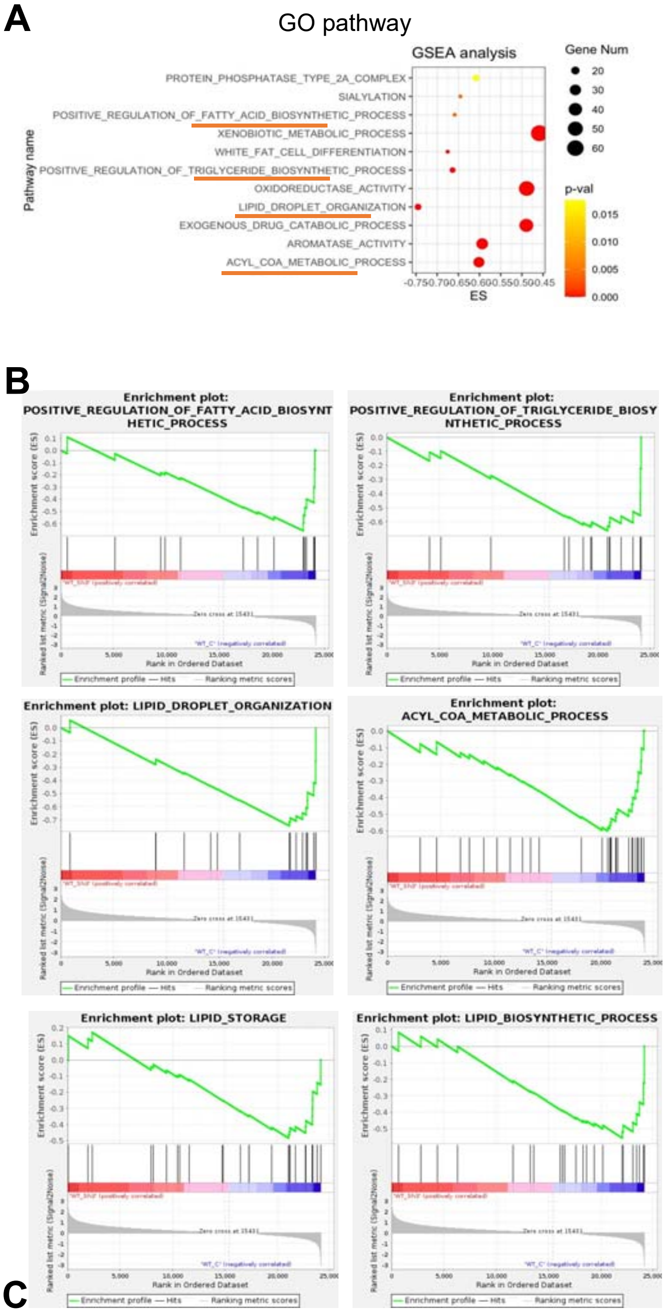

Supplementary Figure 7: FAF2 regulated SREBP1 and its target genes. (A-B) Western blot detected SREBP1, FASN, LPIN protein levels with addition samples. Each line representing an individual mouse liver. The densitometric analysis was provided and ACTIN (beta-ACTIN) or GAPDH was used for loading control. Sample in arrow is the same sample, which was loaded in both gels. (C) Lipase activity assay after treated with ATGL inhibitor. Data are presented as mean ± SEM, *P<0.05; **P<0.01; ****P<0.0001 vs indicated group.

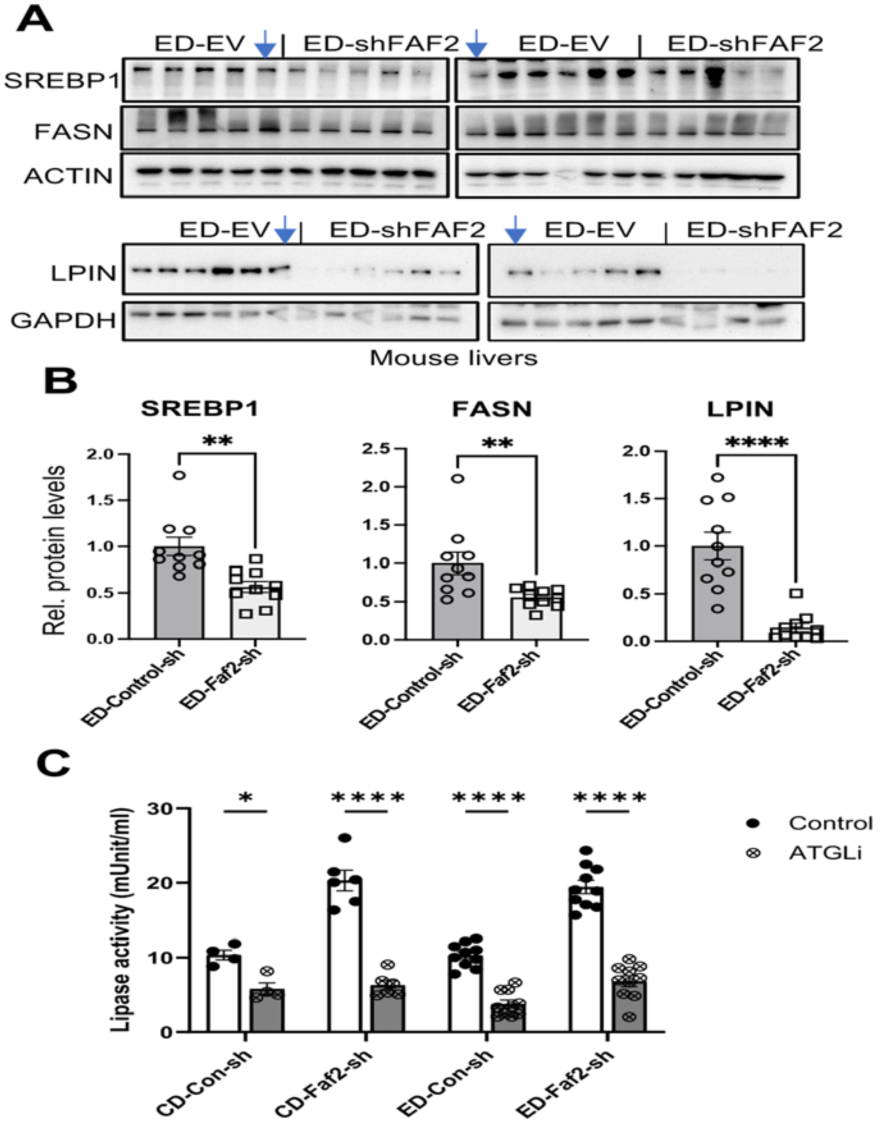

Supplement: Supplementary file 3 [file hc9-9-e0641-s003.pdf]
